# Distributed Closed-Form Multi-UAV Formation Control with Event-Triggered Collision Avoidance and Virtual-Structure Navigation

**DOI:** 10.3390/s26154740

**Published:** 2026-07-26

**Authors:** Wenxue Zhang, Hao Lei, Dušan M. Stipanović

**Affiliations:** 1 School of Mechanical Engineering, Hebei University of Technology, Tianjin 300401, China; wxzhang@hebut.edu.cn (W.Z.); 202521202015@stu.hebut.edu.cn (H.L.); 2 Coordinated Science Laboratory and the Department of Industrial and Enterprise Systems Engineering, University of Illinois at Urbana-Champaign, Urbana, IL 61801, USA

**Keywords:** multi-UAV formation control, decentralized control, collision avoidance, event-triggered control, Lagrangian dynamics, Lyapunov stability

## Abstract

This paper presents a distributed closed-form control strategy for multi-unmanned aerial vehicle (multi-UAV) formation flight in cluttered environments. We propose a virtual-centroid-based architecture that reduces leader dependency while maintaining precise geometric configuration. A virtual-centroid navigation strategy generates reference trajectories for formation coordination. For collision avoidance, a generalized *p*-norm distance function accurately assesses collision risks for diverse obstacle geometries. An event-triggered mechanism incorporating velocity-dependent conditions produces amplitude-modulated, continuity-preserving avoidance vectors. A virtual navigation trajectory dynamically fuses avoidance information with reference tracking, enabling seamless coordination of tracking and collision avoidance. Closed-form controllers are derived for three-degree-of-freedom (3-DoF) dynamic formation control. Safety guarantees and asymptotic convergence in the nominal case are established via generalized Lyapunov analysis for non-smooth dynamical systems. Comparative simulations demonstrate that the proposed method achieves trajectory smoothness comparable to artificial potential field (APF).

## 1. Introduction

Unmanned aerial vehicles (UAVs) have been extensively employed in a wide range of civilian and military applications, including environmental monitoring, precision agriculture, disaster response, target surveillance, and intelligent transportation systems [1,2]. Compared with single-UAV operations, multi-UAV systems provide significant advantages in terms of mission efficiency, spatial coverage, fault tolerance, and operational flexibility [3]. Consequently, coordinated formation flight has become one of the most active research topics in autonomous aerial robotics and cooperative control systems [4].

Existing multi-UAV formation control approaches can generally be categorized into leader–follower methods, behavior-based methods, consensus-based methods, and virtual-structure methods [5,6]. Leader–follower architectures are attractive due to their simple implementation and intuitive command hierarchy; however, they suffer from single-point failure and limited scalability when the leader becomes unavailable or communication links deteriorate [7,8]. Consensus-based approaches improve system robustness by exploiting local information exchange among neighboring agents and have demonstrated strong theoretical guarantees on distributed coordination [9,10]. Virtual-structure methods treat the entire formation as a rigid body, thereby providing superior geometric consistency and trajectory coordination [11,12]. Recent advances have further integrated gradient-free optimization and consensus-based source-seeking strategies to enhance formation adaptability in unknown environments with limited communications [13]. Nevertheless, maintaining formation integrity while simultaneously ensuring collision avoidance in cluttered environments remains a challenging problem, especially when multiple formations operate cooperatively under limited communication bandwidth [14,15].

In practical applications, formation maintenance alone is insufficient because UAVs frequently encounter static obstacles, dynamic obstacles, and inter-agent collision risks [16,17]. Artificial potential field (APF) methods are among the most widely adopted collision avoidance techniques owing to their computational simplicity and real-time implementation capability [18,19]. However, conventional APF approaches are known to suffer from local minima, oscillatory behaviors, and excessive conservatism near obstacle boundaries [20]. To overcome these limitations, numerous optimization-based approaches have been developed. In particular, model predictive control (MPC) and distributed model predictive control (DMPC) frameworks can explicitly incorporate safety constraints and vehicle dynamics into the control design process [21,22]. Despite their effectiveness, these approaches generally require solving online optimization problems at every control step, resulting in increased computational complexity and reduced scalability for large-scale UAV formations [23,24]. Recent studies have also explored game-theoretic methods, velocity obstacle methods, and swarm-intelligence-based strategies to improve obstacle avoidance performance [25]. Nevertheless, achieving computationally efficient and provably safe formation flight remains an open challenge [26].

Recently, Control Barrier Functions (CBFs) have emerged as a powerful mathematical framework for guaranteeing forward invariance of safety sets and enforcing collision-free operation in safety-critical systems [27,28]. By integrating safety constraints directly into the control synthesis process, CBF-based methods provide rigorous collision avoidance guarantees for multi-agent systems and robotic networks [29]. Consequently, CBF theory has been successfully applied to multi-UAV cooperative search, target tracking, and collision avoidance problems [30,31]. However, most existing CBF-based approaches rely on quadratic programming (QP) or MPC-CBF formulations, which still require online optimization and may become computationally expensive as the number of UAVs increases [32]. Safety-critical model predictive control with embedded control barrier functions has been investigated for dynamic obstacle avoidance, yet the centralized optimization bottleneck persists in large-scale deployments [33]. Furthermore, the integration of CBF-inspired safety mechanisms with distributed formation control remains relatively underexplored [34]. Distributed optimization frameworks for multi-agent systems have demonstrated promising scalability properties, though their direct application to formation flight with collision avoidance guarantees is still an active research direction [35]. Recent theoretical advances in autonomous optimization have established global asymptotic stability guarantees for feedback-based optimization loops, providing rigorous analytical foundations for embedding optimization dynamics within closed-loop control systems [36].

Motivated by the above challenges, this paper proposes a distributed closed-form control framework for multi-UAV formation flight in cluttered environments. A virtual-centroid-based architecture is introduced to eliminate the dependence on a physical leader while preserving formation geometry. A virtual-centroid navigation strategy is employed to generate formation references that ensure trajectory smoothness and coordination efficiency. To enhance safety performance, a generalized *p*-norm distance metric is developed to characterize collision risks associated with heterogeneous obstacles and neighboring UAVs. Furthermore, an event-triggered collision avoidance mechanism is designed to activate avoidance actions only when collision risk is increasing, thereby reducing unnecessary evasive maneuvers and improving trajectory smoothness. Unlike conventional MPC-CBF approaches, the proposed method yields lightweight closed-form control laws without requiring online optimization, making it suitable for real-time implementation in large-scale UAV formations.

The main contributions of this paper can be summarized as follows:

1. A virtual-centroid-based decentralized formation architecture is developed to eliminate leader dependence while maintaining formation geometry.
2. A generalized *p*-norm collision assessment mechanism is proposed to evaluate collision risks associated with both obstacles and neighboring UAVs of various geometric configurations.
3. An event-triggered collision avoidance strategy is designed to reduce conservatism while guaranteeing collision-free operation.
4. A computationally efficient closed-form control law is derived, and rigorous stability and safety properties are established through Lyapunov-based analysis.

The remainder of this paper is organized as follows. Section 2 presents the system model and problem formulation. Section 3 develops the collision avoidance mechanism. Section 4 constructs the virtual navigation trajectory. Section 5 derives the decentralized control law. Section 6 provides the stability analysis. Section 7 presents simulation results and comparative studies. Finally, Section 8 concludes the paper.

## 2. Preliminaries and Problem Formulation

### 2.1. Notation

Throughout this paper, $R^{m\times n}$ denotes the space of m×n real matrices. Bold symbols represent matrices and vectors. The identity matrix of dimension *n* is denoted by $I_{n}$, and $0_{m\times n}$ represents the zero matrix. The symbol “:=” implies definition. The Euclidean norm is denoted by $∥\;\cdot \;∥$. For integers k<n, let $N_{k}^{n}≜\left\{k,k+1,\ldots ,n\right\}$.

### 2.2. UAV Dynamic Model

We analyze a multi-formation system wherein *m* formations track trajectories based on a predefined geometric configuration. Each formation consists of *n* UAVs. Operating within a fixed-altitude environment, UAVs maintain stability in height, roll, and pitch through appropriate inner-loop control. Consequently, we focus on kinematic control in the horizontal plane, reducing the problem to 3-DoF motion planning. Two reference frames are established: the body frame $\left\{B_{i,k}\right\}$ rigidly attached to the *k*-th vehicle, and the earth-fixed inertial frame {E}. The kinematic equations for the *k*-th UAV in the *i*-th formation are given by:

(1) $${\dot{\eta }}_{i,k}:=\left[\begin{array}{c} {\dot{p}}_{i,k}\\ {\dot{\theta }}_{i,k} \end{array}\right]=\left[\begin{array}{cc} R_{i,k}\left(\theta_{i,k}\right) & 0_{2\times 1}\\ 0_{1\times 2} & 1 \end{array}\right]\left[\begin{array}{c} v_{i,k}\\ \omega_{i,k} \end{array}\right],\;\forall i\in N_{1}^{m},\;k\in N_{1}^{n},$$

where $p_{i,k}={\left[x_{i,k},\;y_{i,k}\right]}^{⊤}\in R^{2}$ is the position in {E}, $\theta_{i,k}\in \left(-\pi ,\pi \right]$ is the yaw angle, $v_{i,k}={\left[v_{i,k}^{x},\;v_{i,k}^{y}\right]}^{⊤}\in R^{2}$ is the velocity expressed in $\left\{B_{i,k}\right\}$, and $\omega_{i,k}\in R$ is the yaw rate. The rotation matrix is:

(2) $$R_{i,k}\left(\theta_{i,k}\right)=\left[\begin{array}{cc} \cos\theta_{i,k} & -\sin\theta_{i,k}\\ \sin\theta_{i,k} & \cos\theta_{i,k} \end{array}\right].$$

Denoting the generalized velocity as $\nu_{i,k}={\left[{\left(v_{i,k}\right)}^{⊤},\omega_{i,k}\right]}^{⊤}$ and the generalized coordinate derivative as ${\dot{\eta }}_{i,k}={\left[{\left({\dot{p}}_{i,k}\right)}^{⊤},{\dot{\theta }}_{i,k}\right]}^{⊤}$, the kinematic model (1) admits the compact form

(3) $${\dot{\eta }}_{i,k}=J_{i,k}^{-1}\left(\theta_{i,k}\right)\;\nu_{i,k},\;$$

where the Jacobian matrix $J_{i,k}\left(\theta_{i,k}\right)\in R^{3\times 3}$ mapping the inertial-frame rate ${\dot{\eta }}_{i,k}$ to the body-frame velocity $\nu_{i,k}$ is defined as

(4) $$J_{i,k}\left(\theta_{i,k}\right)=\left[\begin{array}{cc} R_{i,k}^{⊤}\left(\theta_{i,k}\right) & 0_{2\times 1}\\ 0_{1\times 2} & 1 \end{array}\right].\;$$

Because $R_{i,k}\left(\theta_{i,k}\right)$ satisfies $R_{i,k}^{⊤}R_{i,k}=I_{2}$ and $\det(R_{i,k})=+1$ for all $\theta_{i,k}\in R$, the Jacobian is orthonormal with

(5) $$J_{i,k}^{-1}\left(\theta_{i,k}\right)=J_{i,k}^{⊤}\left(\theta_{i,k}\right)=\left[\begin{array}{cc} R_{i,k}\left(\theta_{i,k}\right) & 0_{2\times 1}\\ 0_{1\times 2} & 1 \end{array}\right],\;\det\left(J_{i,k}\right)=+1,$$

and consequently

(6) $$J_{i,k}^{-⊤}\left(\theta_{i,k}\right)≜{\left(J_{i,k}^{-1}\left(\theta_{i,k}\right)\right)}^{⊤}=J_{i,k}\left(\theta_{i,k}\right).$$

Hence, $J_{i,k}$ is globally invertible for all yaw angles $\theta_{i,k}\in \left(-\pi ,\pi \right]$ (indeed for all $\theta_{i,k}\in R$), and both $J_{i,k}$ and $J_{i,k}^{-1}$ are uniformly bounded because their entries are trigonometric functions of $\theta_{i,k}$.

The body-fixed Lagrangian dynamics reads

(7) $$M_{i,k}{\dot{\nu }}_{i,k}+C_{i,k}\left(\nu_{i,k}\right)\nu_{i,k}+D_{i,k}\left(\nu_{i,k}\right)\nu_{i,k}+g_{i,k}\left(\eta_{i,k}\right)=\tau_{i,k}+d_{i,k},\;$$

where $M_{i,k}≻0$ is the inertia matrix, $C_{i,k}$ is the Coriolis/centripetal matrix, $D_{i,k}$ is the damping matrix, $g_{i,k}\left(\eta_{i,k}\right)$ is the restoring (e.g., gravitational) term, and $\tau_{i,k}={\left[{\left(u_{i,k}^{v}\right)}^{⊤},u_{i,k}^{\omega }\right]}^{⊤}$ is the physical control input vector. $d_{i,k}$ is the disturbance vector, which satisfies $∥d_{i,k}^{\eta }∥\leq {\bar{d}}_{i,k}$ as $J^{-⊤}$ is uniformly bounded for all $\theta_{i,k}\in \left(-\pi ,\pi \right]$.

Pre-multiplying (7) by $J^{-⊤}$ and substituting (1) yields the earth-fixed equivalent

(8) $$M_{i,k}^{\eta }\left(\eta_{i,k}\right){\ddot{\eta }}_{i,k}+C_{i,k}^{\eta }\left(\nu_{i,k},\eta_{i,k}\right){\dot{\eta }}_{i,k}+D_{i,k}^{\eta }\left(\nu_{i,k},\eta_{i,k}\right){\dot{\eta }}_{i,k}+g_{i,k}^{\eta }\left(\eta_{i,k}\right)=\tau_{i,k}^{\eta }+d_{i,k}^{\eta },\;$$

where the transformed matrices and vectors are defined by matching term-by-term:

(9) $$\begin{array}{cccc} M_{i,k}^{\eta }\left(\eta_{i,k}\right) & =J^{-⊤}M_{i,k}J^{-1},\; & \;C_{i,k}^{\eta }\left(\nu_{i,k},\eta_{i,k}\right) & =J^{-⊤}\left[C_{i,k}-M_{i,k}J^{-1}\dot{J}\right]J^{-1},\\ D_{i,k}^{\eta }\left(\nu_{i,k},\eta_{i,k}\right) & =J^{-⊤}D_{i,k}J^{-1},\; & \;g_{i,k}^{\eta }\left(\eta_{i,k}\right) & =J^{-⊤}g_{i,k}\left(\eta_{i,k}\right),\\ \tau_{i,k}^{\eta } & =J^{-⊤}\tau_{i,k},\; & \;d_{i,k}^{\eta } & =J^{-⊤}d_{i,k}. \end{array}$$

### 2.3. Virtual Centroid and Formation Geometry

In multi-UAV cooperative missions, the formation system must simultaneously accomplish collective navigation toward mission targets and local geometric preservation among team members. To avoid the single-point-of-failure vulnerabilities inherent in leader–follower architectures and the computational burden of fully centralized coordination, we adopt a virtual-centroid-based abstraction that decouples the formation-level navigation from the agent-level configuration maintenance [37,38].

#### 2.3.1. Virtual Centroid and Rigid-Body Formation Mapping

Consider a formation consisting of *n* UAVs with planar positions $p_{i,k}\in R^{2}$, $k\in N_{1}^{n}$. The virtual centroid of the *i*-th formation is defined as the geometric center

(10) $$p_{i}^{c}=\frac{1}{n}\sum_{k=1}^{n}p_{i,k},\;$$

which serves as a reference point representing the collective position of the formation in the task space. Crucially, $p_{i}^{c}$ is a purely mathematical abstraction that does not correspond to any physical vehicle, thereby eliminating the single-point-of-failure risk associated with designated leader UAVs.

**Remark** **1** (On the Computation of $p_{i}^{c}$)**.** *Equation (10) presumes that either (i) a centralized coordinator or a designated formation agent computes the average and broadcasts it to all members, or (ii) all-to-all intra-formation communication is available. In scenarios where agents only communicate with local neighbors, $p_{i}^{c}$ can be replaced by a dynamic average consensus estimate. The control laws derived in Section 5 and Section 6 remain valid provided the estimation error is uniformly bounded and converges sufficiently fast.*

The desired position and orientation of each follower UAV are specified relative to the virtual centroid through the rigid-body transformation

(11) $$\left\{\begin{array}{c} p_{i,k}^{d}=p_{i}^{c}+T_{i}\left(\theta_{i}^{c}\right)\;\delta_{i,k},\\ \theta_{i,k}^{d}=\theta_{i}^{c}+\psi_{i,k}, \end{array}\right.\;\forall i\in N_{1}^{m},\;k\in N_{2}^{n},\;$$

where $\delta_{i,k}\in R^{2}$ is the nominal relative position vector, $\psi_{i,k}\in \left(-\pi ,\pi \right]$ is the nominal relative orientation offset, $\theta_{i}^{c}$ denotes the virtual-centroid yaw angle, and $T_{i}\left(\theta_{i}^{c}\right))$ is the rotation matrix mapping body-relative coordinates to the inertial frame. The set of relative configuration parameters $F_{i}={\left\{\left(\delta_{i,k},\psi_{i,k}\right)\right\}}_{k=1}^{n}$ uniquely determines the formation geometry. By selecting different parameter sets, various formation patterns, including line, wedge, diamond, and ring configurations, can be realized without altering the underlying control structure.

**Figure 1:**
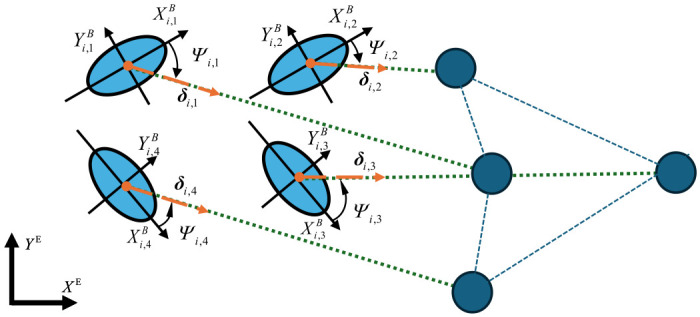
Virtual-centroid-based formation geometry. The left panel illustrates four UAVs (ellipses with body frames $\left\{B_{i,k}\right\}$) and their nominal relative position vectors $\delta_{i,k}$ and orientation offsets $\psi_{i,k}$ with respect to the virtual centroid. The right panel shows the desired formation geometry generated from the relative offsets via the rigid-body transformation (11).

Figure 1 depicts the proposed architecture. The desired formation geometry is induced by the relative position vectors $\delta_{i,k}$ and remains invariant under the centroid rotation $T_{i}\left(\theta_{i}^{c}\right)$, ensuring rigid-body-like formation coherence during maneuvering.

#### 2.3.2. Formation Navigation and Configuration Maintenance

The virtual-centroid representation naturally separates the formation motion into two complementary components: a navigation component that governs the collective translation and rotation of the virtual centroid $p_{i}^{c}\left(t\right)$, and a configuration-maintenance component that preserves the rigid-body offset relationship $p_{i,k}-p_{i}^{c}=T_{i}\left(\theta_{i}^{c}\right)\delta_{i,k}$. This decoupling allows the lower-layer local controllers to focus on tracking performance and collision avoidance while treating the virtual-centroid trajectory as an exogenous reference signal. The specific mechanism for generating $p_{i}^{c}\left(t\right)$ will be addressed in Section 4, where a virtual navigation trajectory is constructed to seamlessly coordinate formation tracking with event-triggered collision avoidance.

### 2.4. Problem Statement

**Problem** **1** (Collision-Free Formation Control)**.** *Derive closed-form control inputs $v_{i,k}$ and $\omega_{i,k}$ for all UAVs based on the kinematic model (1), such that the following objectives are simultaneously satisfied:*

*1.*  *Formation Convergence: Position errors asymptotically converge to zero:*
  (12) $$\lim_{t\to +\infty }∥p_{i,k}\left(t\right)-p_{i,k}^{d}\left(t\right)∥=0,\;\forall i,k.\;$$
*2.*  *Collision Avoidance: Inter-agent and agent–obstacle safety margins remain strictly positive for all time:*
  (13) $$\varphi_{i,k\leftarrow j,l}\left(t\right)>\gamma_{i,k\leftarrow j,l},\;\varphi_{i,k\leftarrow o}\left(t\right)>\gamma_{i,k\leftarrow o},\;\forall t>0,\;\forall i,k,j,l,o.$$

### 2.5. Standard Assumptions

The subsequent stability analysis relies on the following standard assumptions, which are collectively invoked in Theorem 1.

**Assumption** **1** (Bounded Inertia and Damping)**.** *The inertia matrix $M_{i,k}$ and the damping matrix $D_{i,k}$ are uniformly bounded and satisfy $\underline{m}I_{3}⪯M_{i,k}⪯\bar{m}I_{3}$ with $\underline{m}$, $\bar{m}>0$. The disturbance vector $d_{i,k}$ is bounded as $∥d_{i,k}∥\leq {\bar{d}}_{i,k}$ for a known constant ${\bar{d}}_{i,k}>0$.*

**Assumption** **2** (Structural Properties)**.** *The transformed earth-fixed inertia matrix $M_{i,k}^{\eta }\left(\eta_{i,k}\right)$ and Coriolis matrix $C_{i,k}^{\eta }$ satisfy the skew-symmetry condition: ${\dot{M}}_{i,k}^{\eta }-2C_{i,k}^{\eta }$ is skew-symmetric. Moreover, the damping dissipation is positive semi-definite, i.e., ${\tilde{\nu }}_{i,k}^{⊤}D_{i,k}{\tilde{\nu }}_{i,k}\geq 0$ for all ${\tilde{\nu }}_{i,k}$.*

**Assumption** **3.** *The avoidance function $V_{i,k\leftarrow j,l}^{a}$ defined in (16) is continuously differentiable ($C^{1}$) with locally Lipschitz gradients on its support (γ,ψ]. Its time derivative ${\dot{V}}_{i,k\leftarrow j,l}^{a}$ is measurable and bounded along trajectories.*

**Assumption** **4** (Initial Safety)**.** *At t=0, all inter-agent and agent–obstacle distances strictly exceed their respective safety margins:*

(14) $$\varphi_{i,k\leftarrow j,l}\left(0\right)>\gamma_{i,k\leftarrow j,l},\;\varphi_{i,k\leftarrow o}\left(0\right)>\gamma_{i,k\leftarrow o},\;\forall \left(i,k\right),\left(j,l\right),o.$$

**Assumption** **5** (Bounded Initial Energy)**.** *The initial value of the composite Lyapunov function (37) is finite, i.e., V(0)<+∞, which holds whenever Assumption 4 is satisfied and the initial velocity errors ${\tilde{\nu }}_{i,k}\left(0\right)$ are bounded.*

## 3. Less-Conservative Collision Avoidance

### 3.1. Generalized p-Norm Distance Function

Traditional Euclidean distance cannot accurately characterize collision risks for non-circular obstacles, often leading to either excessive conservatism or unsafe risk underestimation. To address this limitation, we introduce a generalized distance metric defined as

(15) $$\varphi_{i,k\leftarrow j,l}={\left(\vartheta_{i,k\leftarrow j,l}^{⊤}\Phi_{i,k\leftarrow j,l}\vartheta_{i,k\leftarrow j,l}\right)}^{\frac{1}{p}},\;$$

where $\vartheta_{i,k\leftarrow j,l}={\left(|x_{j,l}-x_{i,k}{|}^{p/2},{\left|y_{j,l}-y_{i,k}\right|}^{p/2}\right)}^{⊤}$, p>0 is a shape curvature parameter, and $\Phi_{i,k\leftarrow j,l}=\mathrm{diag}\left(\phi_{1},\phi_{2}\right)\in R^{2\times 2}$ denotes a positive definite diagonal matrix that adjusts the aspect ratio and orientation of the distance contour. Restricting Φ to be diagonal ensures that (15) strictly generalizes the weighted *p*-norm ${\left(\phi_{1}{\left|\Delta x\right|}^{p}+\phi_{2}{\left|\Delta y\right|}^{p}\right)}^{1/p}$, preserves homogeneity, and enables rigorous geometric characterization of the resulting level sets.

**Remark** **2** (Generalized Distance Adaptability)**.** *The proposed distance function (15) is adaptable to various obstacle geometries: circular obstacles (p=2, $\Phi =I_{2}$), elliptical obstacles (p=2, $\Phi \neq I_{2}$), rounded rectangles (p>2), rhombic obstacles (p=1), and non-convex star-shaped obstacles (0<p<1).*

### 3.2. Collision Risk Assessment Function

The forbidden set Ω is partitioned into inter-UAV collision set $\bar{\Omega }$ and obstacle collision set $\hat{\Omega }$. Two complementary safety constructs are employed. The collision risk assessment function is defined as a smooth reciprocal-power function:

(16) $$V_{i,k\leftarrow j,l}^{a}=\left\{\begin{array}{cc} {\left(\frac{\psi_{i,k\leftarrow j,l}-\varphi_{i,k\leftarrow j,l}}{\varphi_{i,k\leftarrow j,l}-\gamma_{i,k\leftarrow j,l}}\right)}^{\;\mu },\; & \varphi_{i,k\leftarrow j,l}\in \left(\gamma_{i,k\leftarrow j,l},\;\psi_{i,k\leftarrow j,l}\right]\\ 0,\; & \varphi_{i,k\leftarrow j,l}>\psi_{i,k\leftarrow j,l} \end{array}\right.\;$$

where μ≥2 is a shaping exponent. This construction guarantees $V^{a}\in C^{1}\left(R^{2},R_{\geq 0}\right)$ with locally Lipschitz gradients and compact support in (γ,ψ], satisfying Assumption 3. The gradient follows directly by the chain rule:

(17) $$\frac{\partial V_{i,k\leftarrow j,l}^{a}}{\partial p_{i,k}}=-\mu \;\frac{\left(\psi_{i,k\leftarrow j,l}-\gamma_{i,k\leftarrow j,l}\right)\;{\left(\psi_{i,k\leftarrow j,l}-\varphi_{i,k\leftarrow j,l}\right)}^{\mu -1}}{{\left(\varphi_{i,k\leftarrow j,l}-\gamma_{i,k\leftarrow j,l}\right)}^{\mu +1}}\;\frac{\partial \varphi_{i,k\leftarrow j,l}}{\partial p_{i,k}},\;$$

which points in the strict repulsive direction and vanishes continuously at $\varphi =\psi_{i,k\leftarrow j,l}$.

### 3.3. Velocity-Informed Event-Triggered Mechanism

To reduce conservatism and avoid Zeno behavior, avoidance is triggered only when the time derivative of risk is positive (${\dot{V}}_{i,k\leftarrow j,l}^{a}>0$), indicating increasing danger. The avoidance vector comprises two complementary components.

Continuity Control (smooth switching):

(18) $$E_{i,k\leftarrow j,l}^{cc}=h^{cc}\left(1-\frac{{\dot{V}}_{i,k\leftarrow j,l}^{a}}{\alpha_{i,k\leftarrow j,l}}\right){\left(\frac{\partial V_{i,k\leftarrow j,l}^{a}}{\partial p_{i,k}}\right)}^{⊤},\;$$

where $h^{cc}\left(\;\cdot \;\right)$ is a smooth $C^{1}$ step function satisfying $h^{cc}\left(s\right)=0$ for s≤0, $h^{cc}\left(s\right)=1$ for s≥1, and $0<h^{cc}\left(s\right)<1$ for s∈(0,1), with bounded derivative; $\alpha_{i,k\leftarrow j,l}<0$ is the activation threshold.

Amplitude Control (event-triggered):

(19) $$E_{i,k\leftarrow j,l}^{ac}=h^{ac}\left({\dot{V}}_{i,k\leftarrow j,l}^{a}\right){\left(\frac{\partial V_{i,k\leftarrow j,l}^{a}}{\partial p_{i,k}}\right)}^{⊤},\;$$

where $h^{ac}\left(x\right)=\max\left(0,x\right)$ is the standard ReLU function (Lipschitz continuous with constant 1).

The composite avoidance vector is

(20) $$E_{i,k\leftarrow j,l}^{a}=c_{i,k\leftarrow j,l}^{cc}E_{i,k\leftarrow j,l}^{cc}+c_{i,k\leftarrow j,l}^{ac}E_{i,k\leftarrow j,l}^{ac},\;$$

where $c_{i,k\leftarrow j,l}^{cc},c_{i,k\leftarrow j,l}^{ac}>0$ are tunable blending weights.

The overall avoidance vector for UAV (i,k) aggregates all threats:

(21) $$E_{i,k}^{a}=\sum_{\begin{array}{c} l=1\\ l\neq k \end{array}}^{n}E_{i,k\leftarrow i,l}^{a}+\sum_{\begin{array}{c} j=1\\ j\neq i \end{array}}^{m}\sum_{l=1}^{n}E_{i,k\leftarrow j,l}^{a}+\sum_{o=1}^{\bar{m}}E_{i,k\leftarrow o}^{a}.\;$$

**Figure 2:**
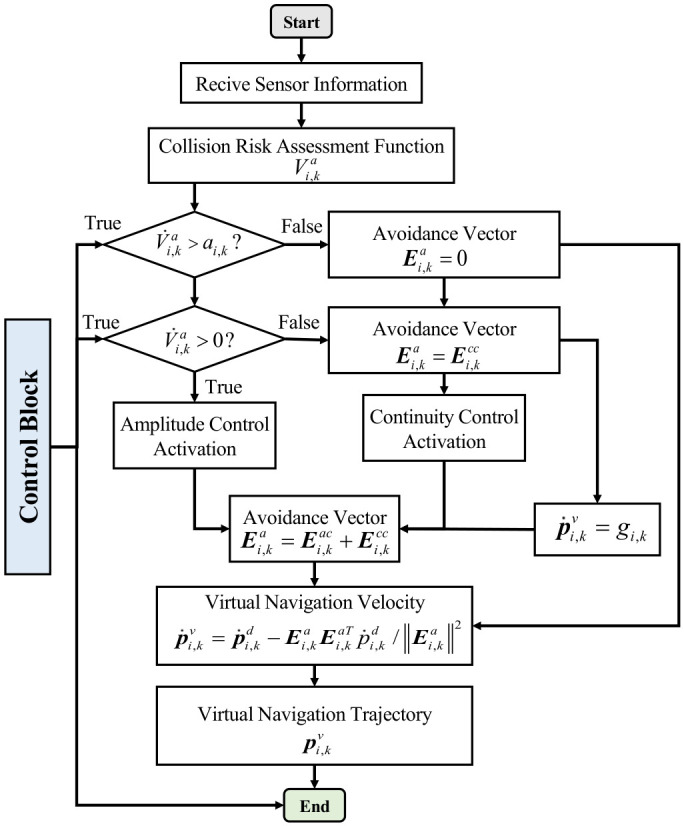
Flowchart of the proposed event-triggered collision avoidance and virtual navigation strategy. The diagram illustrates the real-time execution pipeline: collision risk assessment via $V_{i,k}^{a}$, velocity-informed event-triggered activation of continuity and amplitude controls, composite avoidance vector synthesis, and virtual navigation velocity generation. When $∥E_{i,k}^{a}∥=0$, the virtual navigation velocity defaults to the smooth transition function $g_{i,k}\left(t\right)$; otherwise, the desired trajectory is projected onto the collision-free subspace via (23).

The complete real-time execution pipeline of the proposed velocity-informed event-triggered mechanism is summarized in Figure 2. Upon receiving sensor information, the collision risk assessment function $V_{i,k}^{a}$ is evaluated. Depending on the sign of ${\dot{V}}_{i,k}^{a}$ relative to the threshold $\alpha_{i,k}$, the system either deactivates avoidance ($E_{i,k}^{a}=0$), activates only the continuity control ($E_{i,k}^{cc}$), or engages both continuity and amplitude controls ($E_{i,k}^{a}=E_{i,k}^{ac}+E_{i,k}^{cc}$). The resulting avoidance vector dynamically corrects the virtual navigation velocity ${\dot{p}}_{i,k}^{v}$ via perpendicular projection, yielding the auxiliary trajectory $p_{i,k}^{v}$ for the subsequent control synthesis.

## 4. Virtual Navigation Trajectory

### 4.1. Desired Trajectory Decomposition

Traditional strategies freeze desired trajectories during avoidance, compromising tracking performance and mission efficiency. We decompose the desired trajectory derivative into components parallel and perpendicular to the avoidance vector:

(22) $${\dot{p}}_{i,k}^{d⊥}={\dot{p}}_{i,k}^{d}-\frac{E_{i,k}^{a}{\left(E_{i,k}^{a}\right)}^{⊤}}{{∥E_{i,k}^{a}∥}^{2}}{\dot{p}}_{i,k}^{d}.\;$$

The perpendicular component ${\dot{p}}_{i,k}^{d⊥}$ maintains tracking progress while avoiding collision-prone motion along the threat direction.

### 4.2. Virtual Trajectory Correction

The virtual navigation velocity is synthesized by projecting the desired trajectory onto the collision-free subspace:

(23) $${\dot{p}}_{i,k}^{v}=\left\{\begin{array}{cc} {\dot{p}}_{i,k}^{d⊥}, & ∥E_{i,k}^{a}∥\neq 0\\ g_{i,k}\left(t\right), & ∥E_{i,k}^{a}∥=0, \end{array}\right.\;$$

where $g_{i,k}\left(t\right)$ is a smooth transition function ensuring exponential convergence of $p_{i,k}^{v}$ to $p_{i,k}^{d}$ when avoidance is inactive. The specific form of $g_{i,k}\left(t\right)$ does not impact the stability analysis, as shown in Theorem 1. In practice, the auxiliary trajectory $p_{i,k}^{v}\left(t\right)$ can be generated in real-time using Algorithm 1.

The virtual navigation trajectory is obtained by temporal integration:

(24) $$p_{i,k}^{v}\left(t\right)=p_{i,k}^{d}\left(0\right)+\int_{0}^{t}{\dot{p}}_{i,k}^{v}\left(\tau \right)\;d\tau .\;$$

In Algorithm 1, $\tau_{in}$ denotes the triggering instant of the avoidance maneuver.
**Algorithm 1** Virtual Navigation Trajectory Generation  1:Initialize $p_{i,k}^{v}\left(t\right)=p_{i,k}^{d}\left(t\right)$ for t≥0  2:**while** collision avoidance occurs ($∥E_{i,k}^{a}\left(\tau_{in}\right)∥\neq 0$) **do**  3:      Set $p_{i,k}^{v}\left(t\right)=p_{i,k}^{d}\left(\tau_{in}\right)+\int_{\tau_{in}}^{t}{\dot{p}}_{i,k}^{v}\left(\tau \right)\;d\tau$  4:      Compute ${\dot{p}}_{i,k}^{v}$ via (23)  5:**end while**  6:Update $p_{i,k}^{v}\left(t\right)$ to latest $p_{i,k}^{d}\left(t\right)$ using smooth transition  7:Repeat when new avoidance is triggered

## 5. Control Law Design

The control architecture follows the signal-flow hierarchy $E\to v^{d}\to \tau$ summarized in Table 1. It comprises three cascaded layers: (i) an outer-loop guidance layer that fuses formation tracking, collision avoidance, and deadlock escape into a unified guidance vector; (ii) a mid-loop kinematic layer that projects this guidance into feasible body-frame velocity commands; and (iii) an inner-loop dynamic layer that compensates for the full nonlinear dynamics via closed-form feedback. This hierarchical decoupling ensures that the computationally lightweight guidance and kinematic layers operate at high rates, while the dynamic layer handles model-based compensation without requiring online optimization.

Since the closed-form controller derived below is expressed in the earth-fixed frame (cf. (35)), the subsequent stability analysis in Section 6 is carried out entirely in the earth-fixed coordinates. To this end, define the desired generalized state trajectory as

(25) $$\eta_{i,k}^{d}\left(t\right)=\left[\begin{array}{c} p_{i,k}^{v}\left(t\right)\\ \theta_{i,k}^{v}\left(t\right) \end{array}\right],\;{\dot{\eta }}_{i,k}^{d}\left(t\right)=\left[\begin{array}{c} {\dot{p}}_{i,k}^{v}\left(t\right)\\ {\dot{\theta }}_{i,k}^{v}\left(t\right) \end{array}\right],\;$$

where $p_{i,k}^{v}$ and $\theta_{i,k}^{v}$ are generated by the virtual navigation strategy in Section 4. The generalized velocity error is defined as

(26) $${\dot{\tilde{\eta }}}_{i,k}≜{\dot{\eta }}_{i,k}-{\dot{\eta }}_{i,k}^{d}=\left[\begin{array}{c} {\dot{p}}_{i,k}-{\dot{p}}_{i,k}^{v}\\ \omega_{i,k}-{\dot{\theta }}_{i,k}^{v} \end{array}\right],\;$$

which is the sole velocity variable used in the stability proof.

### 5.1. Convergence Vector Synthesis

The composite guidance vector for agent (i,k) is synthesized by superposing three complementary components:

(27) $$E_{i,k}=-E_{i,k}^{o}+E_{i,k}^{a}+E_{i,k}^{\epsilon },\;$$

where $E_{i,k}^{o}$ is the formation objective vector (attractive), $E_{i,k}^{a}$ is the collision avoidance vector (repulsive), and $E_{i,k}^{\epsilon }$ is the deadlock perturbation vector.

**Figure 3:**
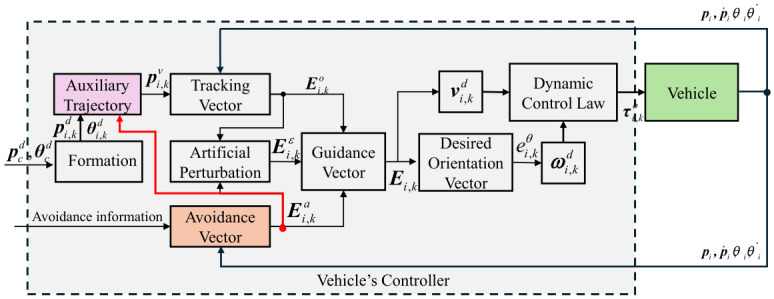
Hierarchical control architecture for the *i*-th UAV in the *k*-th formation. The signal flow follows $E\to v^{d}\to \tau$ (see Table 1). (**Left**): The outer guidance layer synthesizes the composite vector $E_{i,k}=-E_{i,k}^{o}+E_{i,k}^{a}+E_{i,k}^{\epsilon }$. (**Right**): The inner-loop dynamic layer computes the control torque $\tau_{i,k}^{\eta }$ via (35). Red lines indicate the event-triggered avoidance feedback path that dynamically corrects the auxiliary trajectory and injects deadlock perturbation when $∥E_{i,k}^{a}∥\neq 0$.

Figure 3 illustrates the overall control architecture. The outer-loop guidance layer fuses formation tracking ($E_{i,k}^{o}$), collision avoidance ($E_{i,k}^{a}$), and deadlock perturbation ($E_{i,k}^{\epsilon }$) into the composite guidance vector $E_{i,k}$. The mid-loop kinematic layer projects this vector into feasible body-frame velocity commands and subsequently extracts the body-frame linear velocity $v_{i,k}^{d}$ and the desired yaw rate $\omega_{i,k}^{d}$. Finally, the inner-loop dynamic layer closes the loop around the vehicle dynamics (8) via the earth-fixed equivalent control law (35). The red feedback paths highlight the event-triggered avoidance mechanism: when collision risk increases ($∥E_{i,k}^{a}∥\neq 0$), the auxiliary trajectory module dynamically corrects the reference path, while the artificial perturbation module injects orthogonal excitation to escape potential deadlocks.

#### 5.1.1. Formation Objective Vector

Let $e_{i,k}=p_{i,k}-p_{i,k}^{v}$ denote the position tracking error with respect to the virtual navigation trajectory $p_{i,k}^{v}$ defined in (24). A continuously differentiable (almost everywhere), non-negative function $V_{i,k}^{o}\left(e_{i,k}\right)$ is employed, satisfying $\partial V_{i,k}^{o}/\partial e_{i,k}=0$ if and only if $e_{i,k}=0$. The formation objective vector is then defined as the negative gradient:

(28) $$E_{i,k}^{o}={\left(\frac{\partial V_{i,k}^{o}}{\partial e_{i,k}}\right)}^{\;⊤},\;\forall i\in N_{1}^{m},\;k\in N_{1}^{n}.\;$$

This ensures that $E_{i,k}^{o}$ points toward the virtual navigation trajectory (i.e., $E_{i,k}^{o}$ is attractive) when no avoidance is active.

#### 5.1.2. Deadlock Perturbation Vector

A deadlock occurs when the objective vector and the avoidance vector counterbalance each other, i.e., $E_{i,k}^{o}+E_{i,k}^{a}\approx 0$ while $e_{i,k}\neq 0$. To escape such local equilibria, the perturbation vector is injected orthogonally to $E_{i,k}^{a}$:

(29) $$E_{i,k}^{\epsilon }=\left\{\begin{array}{cc} \frac{\mu_{i,k}^{\epsilon }}{∥E_{i,k}^{a}∥}\;\Theta \;\left(\frac{\pi }{2}\right)E_{i,k}^{a}, & \mathrm{if}\;E_{i,k}^{a}\neq 0\;\mathrm{and}\;E_{i,k}^{o}=-E_{i,k}^{a},\\ 0, & \mathrm{otherwise},\; \end{array}\right.$$

where $\mu_{i,k}^{\epsilon }>0$ is a tuning parameter and Θ(π/2) is the planar rotation matrix by π/2, ensuring $E_{i,k}^{\epsilon }⊥E_{i,k}^{a}$.

### 5.2. Control Law Derivation

This vector represents the inertial-frame velocity command issued to the mid-loop kinematic layer. It is subsequently decomposed into the body-frame linear velocity $v_{i,k}^{d}$ and the virtual navigation yaw angle $\theta_{i,k}^{v}$.

We now derive the angle control laws governing the heading dynamics of both leader and follower UAVs. For leader UAVs, the virtual navigation angle is defined as

(30) $$\theta_{i,1}^{v}=\mathrm{atan}2c\left(E_{i,1}^{y},E_{i,1}^{x}\right)\in R,\;\forall i\in N_{1}^{m}.\;$$

Here, atan2c(·,·) denotes the phase-unwrapping variant of atan2(·,·), which resolves phase discontinuities. For follower UAVs, the auxiliary angle is specified by

(31) $$\theta_{i,k}^{v}=\mathrm{atan}2c\left(E_{i,k}^{y},E_{i,k}^{x}\right)+\psi_{i,k},\;\forall i\in N_{1}^{m},k\in N_{2}^{n}.\;$$

Here, $\psi_{i,k}$ serves as a formation parameter for adjusting the heading alignment of follower UAVs.

Define the yaw tracking error

(32) $$e_{i,k}^{\theta }=\theta_{i,k}-\theta_{i,k}^{v},\;$$

where $\theta_{i,k}^{v}$ is the virtual navigation yaw angle.

The disturbance rejection term is designed in the body-fixed frame as

(33) $$\Lambda_{i,k}=\left\{\begin{array}{cc} -{\bar{d}}_{i,k}\;\frac{{\tilde{\nu }}_{i,k}}{∥{\tilde{\nu }}_{i,k}∥}, & \mathrm{if}\;∥{\tilde{\nu }}_{i,k}∥\neq 0,\\ 0, & \mathrm{otherwise}, \end{array}\right.\;$$

with ${\tilde{\nu }}_{i,k}=\nu_{i,k}-J_{i,k}^{-1}\left(\theta_{i,k}\right){\dot{\eta }}_{i,k}^{d}=J_{i,k}^{-1}\left(\theta_{i,k}\right){\dot{\tilde{\eta }}}_{i,k}$, the body-frame velocity error. Its earth-fixed equivalent is

(34) $$\Lambda_{i,k}^{\eta }=J^{-⊤}\left(\theta_{i,k}\right)\Lambda_{i,k}=-{\bar{d}}_{i,k}\;\frac{{\dot{\tilde{\eta }}}_{i,k}}{∥{\dot{\tilde{\eta }}}_{i,k}∥},$$

where the last equality follows from $J^{-⊤}=J$ and the orthonormality of J (cf. (6)).

For rigorous stability analysis, the control law is expressed in the earth-fixed frame. Substituting (1) and its time derivative into the body-fixed dynamics (7), the generalized control input $\tau_{i,k}^{\eta }=J^{-⊤}\left(\theta_{i,k}\right)\tau_{i,k}$ is designed as

(35) $$\begin{array}{cc} \tau_{i,k}^{\eta } & =M_{i,k}^{\eta }\left(\eta_{i,k}\right){\ddot{\eta }}_{i,k}^{d}+C_{i,k}^{\eta }\left({\dot{\eta }}_{i,k},\eta_{i,k}\right){\dot{\eta }}_{i,k}^{d}+D_{i,k}^{\eta }\left({\dot{\eta }}_{i,k},\eta_{i,k}\right){\dot{\eta }}_{i,k}^{d}+g_{i,k}^{\eta }\left(\eta_{i,k}\right)\\ \; & \;-K_{i,k}\left({\dot{\eta }}_{i,k}-{\dot{\eta }}_{i,k}^{d}\right)-\Lambda_{i,k}^{\eta }+\left[\begin{array}{c} E_{i,k}\\ -K_{i,k}^{\omega }\;e_{i,k}^{\theta } \end{array}\right], \end{array}\;$$

where $\eta_{i,k}^{d}$ and ${\dot{\eta }}_{i,k}^{d}$ are given by (25), and $K_{i,k}≻0$ is a positive-definite gain matrix. The term $-\Lambda_{i,k}^{\eta }$ compensates for the matched disturbance $d_{i,k}^{\eta }$ in the sense of (34).

## 6. Stability Analysis

### 6.1. Preliminaries for Stability Proof

The stability analysis builds upon Assumption 1 and the structural properties established in Section 2.2. No additional assumptions are introduced beyond those stated in Section 2.5. Because the controller (35) is formulated entirely in the earth-fixed coordinates, the Lyapunov argument below is carried out in the η-frame using the generalized velocity error ${\dot{\tilde{\eta }}}_{i,k}$ defined in (26).

The following restatement collects the properties required for the Lyapunov argument. Under Assumption 2, the transformed earth-fixed inertia matrix $M_{i,k}^{\eta }\left(\eta_{i,k}\right)$ and Coriolis matrix $C_{i,k}^{\eta }$ satisfy the skew-symmetry condition

(36) $${\dot{\tilde{\eta }}}_{i,k}^{⊤}\left({\dot{M}}_{i,k}^{\eta }-2C_{i,k}^{\eta }\right){\dot{\tilde{\eta }}}_{i,k}=0,\;\;\forall {\dot{\tilde{\eta }}}_{i,k},\;$$

and the damping dissipation is positive semi-definite, i.e., ${\dot{\tilde{\eta }}}_{i,k}^{⊤}D_{i,k}^{\eta }{\dot{\tilde{\eta }}}_{i,k}\geq 0$ for all ${\dot{\tilde{\eta }}}_{i,k}$.

### 6.2. Lyapunov Function Construction

Consider the composite Lyapunov function candidate for the entire multi-formation system:

(37) $$\begin{array}{c} \;V=\sum_{i=1}^{m}\sum_{k=1}^{n}V_{i,k}^{o}\left(e_{i,k}\right)+\frac{1}{2}\sum_{i=1}^{m}\sum_{k=1}^{n}K_{i,k}^{\omega }{\left(e_{i,k}^{\theta }\right)}^{2}+\frac{1}{2}\sum_{i=1}^{m}\sum_{k=1}^{n}\sum_{\begin{array}{c} l=1\\ l\neq k \end{array}}^{n}c_{i,k\leftarrow i,l}^{cc}V_{i,k\leftarrow i,l}^{a}\\ \;+\frac{1}{2}\sum_{i=1}^{m}\sum_{k=1}^{n}\sum_{\begin{array}{c} j=1\\ j\neq i \end{array}}^{m}\sum_{l=1}^{n}c_{i,k\leftarrow j,l}^{cc}V_{i,k\leftarrow j,l}^{a}+\sum_{i=1}^{m}\sum_{k=1}^{n}\sum_{o=1}^{\bar{m}}V_{i,k\leftarrow o}^{a}+\frac{1}{2}\sum_{i=1}^{m}\sum_{k=1}^{n}{\dot{\tilde{\eta }}}_{i,k}^{⊤}M_{i,k}^{\eta }{\dot{\tilde{\eta }}}_{i,k}. \end{array}$$

The function *V* is positive definite with respect to $\left(e_{i,k},e_{i,k}^{\theta },{\dot{\tilde{\eta }}}_{i,k}\right)$ and radially unbounded within the safe set. Under Assumptions 3 and 5, V(0) is finite and positive. Each term in (37) is continuously differentiable almost everywhere, and *V* is proper (level sets are compact) in the interior of the safe set.

### 6.3. Main Stability Theorem

**Theorem** **1** (Collision-free formation convergence)**.** *Consider the multi-UAV multi-formation system governed by (8) under the control law (35) with the guidance vector (27) and the virtual navigation trajectory (23) and (24). Suppose Assumptions 1–5 hold. Then, the following properties are guaranteed:*

*(P1)*  *Safety (Collision Avoidance): No collisions occur for all t≥0, i.e., *
  (38) $$\varphi_{i,k\leftarrow j,l}\left(t\right)>\gamma_{i,k\leftarrow j,l},\;\varphi_{i,k\leftarrow o}\left(t\right)>\gamma_{i,k\leftarrow o},\;\forall t\geq 0,\;\forall \left(i,k\right),\left(j,l\right),o.\;$$
*(P2)*  *Position Convergence: In the disturbance-free nominal case (${\bar{d}}_{i,k}=0$), the position errors converge asymptotically to zero:*
  (39) $$\lim_{t\to \infty }∥p_{i,k}\left(t\right)-p_{i,k}^{v}\left(t\right)∥=0,\;\forall i,k.\;$$

### 6.4. Proof of Theorem 1

The proof follows a Lyapunov-based invariance analysis. We evaluate the time derivative of *V* along the closed-loop trajectories, establish non-positivity, prove collision avoidance by contradiction, and conclude convergence via invariant set arguments.

#### 6.4.1. Stage 1: Time Derivative of *V*

Differentiating (37) along the closed-loop trajectories yields

(40) $$\begin{array}{c} \;\dot{V}=\sum_{i=1}^{m}\sum_{k=1}^{n}\frac{\partial V_{i,k}^{o}}{\partial p_{i,k}}\left({\dot{p}}_{i,k}-{\dot{p}}_{i,k}^{v}\right)+\sum_{i=1}^{m}\sum_{k=1}^{n}K_{i,k}^{\omega }e_{i,k}^{\theta }\left(\omega_{i,k}-{\dot{\theta }}_{i,k}^{v}\right)+\sum_{i=1}^{m}\sum_{k=1}^{n}\sum_{\begin{array}{c} l=1\\ l\neq k \end{array}}^{n}c_{i,k\leftarrow i,l}^{cc}\frac{\partial V_{i,k\leftarrow i,l}^{a}}{\partial p_{i,k}}{\dot{p}}_{i,k}\\ \;+\sum_{i=1}^{m}\sum_{k=1}^{n}\sum_{\begin{array}{c} j=1\\ j\neq i \end{array}}^{m}\sum_{l=1}^{n}c_{i,k\leftarrow j,l}^{cc}\frac{\partial V_{i,k\leftarrow j,l}^{a}}{\partial p_{i,k}}{\dot{p}}_{i,k}+\sum_{i=1}^{m}\sum_{k=1}^{n}\sum_{o=1}^{\bar{m}}\frac{\partial V_{i,k\leftarrow o}^{a}}{\partial p_{i,k}}{\dot{p}}_{i,k}+\sum_{i=1}^{m}\sum_{k=1}^{n}\Theta_{i,k}, \end{array}$$

where the kinetic term $\Theta_{i,k}$ is the time derivative of the last term in (37):

(41) $$\Theta_{i,k}={\dot{\tilde{\eta }}}_{i,k}^{⊤}M_{i,k}^{\eta }{\ddot{\tilde{\eta }}}_{i,k}+\frac{1}{2}{\dot{\tilde{\eta }}}_{i,k}^{⊤}{\dot{M}}_{i,k}^{\eta }{\dot{\tilde{\eta }}}_{i,k}=\frac{d}{dt}\left(\frac{1}{2}{\dot{\tilde{\eta }}}_{i,k}^{⊤}M_{i,k}^{\eta }{\dot{\tilde{\eta }}}_{i,k}\right).\;$$

#### 6.4.2. Stage 2: Evaluation of the Kinetic Term $\Theta_{i,k}$

Substituting the earth-fixed control law (35) into the dynamics (8) and subtracting the desired acceleration term yields the closed-loop error equation

(42) $$M_{i,k}^{\eta }{\ddot{\tilde{\eta }}}_{i,k}+C_{i,k}^{\eta }{\dot{\tilde{\eta }}}_{i,k}+D_{i,k}^{\eta }{\dot{\tilde{\eta }}}_{i,k}=-K_{i,k}{\dot{\tilde{\eta }}}_{i,k}-\Lambda_{i,k}^{\eta }+u_{c,i,k}+d_{i,k}^{\eta },\;$$

where the Coriolis and damping terms have been grouped on the left-hand side by exploiting the structural linearity of $C_{i,k}^{\eta }$ and $D_{i,k}^{\eta }$ in ${\dot{\eta }}_{i,k}$, and the guidance-related input is defined as

(43) $$u_{c,i,k}≜\left[\begin{array}{c} E_{i,k}\\ -K_{i,k}^{\omega }e_{i,k}^{\theta } \end{array}\right].$$

Substituting (42) into (41) gives

$$\begin{array}{cc} \Theta_{i,k} & ={\dot{\tilde{\eta }}}_{i,k}^{⊤}\left[-C_{i,k}^{\eta }{\dot{\tilde{\eta }}}_{i,k}-D_{i,k}^{\eta }{\dot{\tilde{\eta }}}_{i,k}-K_{i,k}{\dot{\tilde{\eta }}}_{i,k}-\Lambda_{i,k}^{\eta }+u_{c,i,k}+d_{i,k}^{\eta }\right]+\frac{1}{2}{\dot{\tilde{\eta }}}_{i,k}^{⊤}{\dot{M}}_{i,k}^{\eta }{\dot{\tilde{\eta }}}_{i,k}\\ \; & ={\dot{\tilde{\eta }}}_{i,k}^{⊤}\left(\frac{1}{2}{\dot{M}}_{i,k}^{\eta }-C_{i,k}^{\eta }\right){\dot{\tilde{\eta }}}_{i,k}-{\dot{\tilde{\eta }}}_{i,k}^{⊤}D_{i,k}^{\eta }{\dot{\tilde{\eta }}}_{i,k}-{\dot{\tilde{\eta }}}_{i,k}^{⊤}K_{i,k}{\dot{\tilde{\eta }}}_{i,k}+{\dot{\tilde{\eta }}}_{i,k}^{⊤}u_{c,i,k}+{\dot{\tilde{\eta }}}_{i,k}^{⊤}\left(d_{i,k}^{\eta }-\Lambda_{i,k}^{\eta }\right).\; \end{array}$$

By the skew-symmetry property (36), the first term vanishes identically, yielding the exact expression

(44) $$\Theta_{i,k}=-{\dot{\tilde{\eta }}}_{i,k}^{⊤}D_{i,k}^{\eta }{\dot{\tilde{\eta }}}_{i,k}-{\dot{\tilde{\eta }}}_{i,k}^{⊤}K_{i,k}{\dot{\tilde{\eta }}}_{i,k}+{\dot{\tilde{\eta }}}_{i,k}^{⊤}u_{c,i,k}+{\dot{\tilde{\eta }}}_{i,k}^{⊤}\left(d_{i,k}^{\eta }-\Lambda_{i,k}^{\eta }\right).\;$$

Summing over all agents and exploiting the damping dissipation ${\dot{\tilde{\eta }}}_{i,k}^{⊤}D_{i,k}^{\eta }{\dot{\tilde{\eta }}}_{i,k}\geq 0$ (Assumption 2), we obtain

(45) $$\sum_{i=1}^{m}\sum_{k=1}^{n}\Theta_{i,k}\leq -\sum_{i=1}^{m}\sum_{k=1}^{n}{\dot{\tilde{\eta }}}_{i,k}^{⊤}K_{i,k}{\dot{\tilde{\eta }}}_{i,k}+\sum_{i=1}^{m}\sum_{k=1}^{n}{\dot{\tilde{\eta }}}_{i,k}^{⊤}u_{c,i,k}+\sum_{i=1}^{m}\sum_{k=1}^{n}{\dot{\tilde{\eta }}}_{i,k}^{⊤}\left(d_{i,k}^{\eta }-\Lambda_{i,k}^{\eta }\right).$$

For the disturbance term, by Assumption 1 and the design (34),

(46) $${\dot{\tilde{\eta }}}_{i,k}^{⊤}\left(d_{i,k}^{\eta }-\Lambda_{i,k}^{\eta }\right)\leq ∥{\dot{\tilde{\eta }}}_{i,k}∥\left(∥d_{i,k}^{\eta }∥-{\bar{d}}_{i,k}\right)\leq 0,\;$$

so that

(47) $$\sum_{i=1}^{m}\sum_{k=1}^{n}\Theta_{i,k}\leq -\sum_{i=1}^{m}\sum_{k=1}^{n}{\dot{\tilde{\eta }}}_{i,k}^{⊤}K_{i,k}{\dot{\tilde{\eta }}}_{i,k}+\sum_{i=1}^{m}\sum_{k=1}^{n}{\dot{\tilde{\eta }}}_{i,k}^{⊤}u_{c,i,k}.$$

The first quadratic term provides the desired dissipation. The second term (guidance-related cross-term) is treated separately in Stage 3.

#### 6.4.3. Stage 3: Guidance Term Cancellation

This stage closes the derivation gap by explicitly tracking the guidance cross-term from (47). Using the partition ${\dot{\tilde{\eta }}}_{i,k}={\left[{\left({\dot{p}}_{i,k}-{\dot{p}}_{i,k}^{v}\right)}^{⊤},\;{\tilde{\omega }}_{i,k}\right]}^{⊤}$ with ${\tilde{\omega }}_{i,k}≜\omega_{i,k}-{\dot{\theta }}_{i,k}^{v}$, and expanding the inner product via (43) yields

(48) $$\sum_{i,k}{\dot{\tilde{\eta }}}_{i,k}^{⊤}u_{c,i,k}=\sum_{i,k}{\left({\dot{p}}_{i,k}-{\dot{p}}_{i,k}^{v}\right)}^{⊤}E_{i,k}\;-\sum_{i,k}{\tilde{\omega }}_{i,k}K_{i,k}^{\omega }e_{i,k}^{\theta }.\;$$

From the objective vector definition (28) and the error $e_{i,k}=p_{i,k}-p_{i,k}^{v}$, we have

(49) $$\frac{\partial V_{i,k}^{o}}{\partial e_{i,k}}=E_{i,k}^{o⊤}.\;$$

Because $e_{i,k}=p_{i,k}-p_{i,k}^{v}$ and $\partial e_{i,k}/\partial p_{i,k}=I_{2}$, the chain rule gives

(50) $$\frac{\partial V_{i,k}^{o}}{\partial p_{i,k}}=\frac{\partial V_{i,k}^{o}}{\partial e_{i,k}}\frac{\partial e_{i,k}}{\partial p_{i,k}}=E_{i,k}^{o⊤}.\;$$

Hence, the first term of (40) can be rewritten as

(51) $$\sum_{i=1}^{m}\sum_{k=1}^{n}\frac{\partial V_{i,k}^{o}}{\partial p_{i,k}}\left({\dot{p}}_{i,k}-{\dot{p}}_{i,k}^{v}\right)=\sum_{i=1}^{m}\sum_{k=1}^{n}E_{i,k}^{o⊤}\left({\dot{p}}_{i,k}-{\dot{p}}_{i,k}^{v}\right).\;$$

Substituting the composite guidance vector (27), $E_{i,k}=-E_{i,k}^{o}+E_{i,k}^{a}+E_{i,k}^{\epsilon }$, into the position channel of (48) gives

(52) $$\begin{array}{ccc} \;\sum_{i=1}^{m}\sum_{k=1}^{n}{\left({\dot{p}}_{i,k}-{\dot{p}}_{i,k}^{v}\right)}^{⊤}E_{i,k} & = & -\sum_{i=1}^{m}\sum_{k=1}^{n}E_{i,k}^{o⊤}\left({\dot{p}}_{i,k}-{\dot{p}}_{i,k}^{v}\right) \end{array}$$

(53) $$\begin{array}{ccc} & & \;+\sum_{i=1}^{m}\sum_{k=1}^{n}E_{i,k}^{a⊤}\left({\dot{p}}_{i,k}-{\dot{p}}_{i,k}^{v}\right)+\sum_{i=1}^{m}\sum_{k=1}^{n}E_{i,k}^{\epsilon ⊤}\left({\dot{p}}_{i,k}-{\dot{p}}_{i,k}^{v}\right). \end{array}$$

Adding (51) and the first term of (52) produces the exact cancellation

(54) $$\sum_{i=1}^{m}\sum_{k=1}^{n}E_{i,k}^{o⊤}\left({\dot{p}}_{i,k}-{\dot{p}}_{i,k}^{v}\right)-\sum_{i=1}^{m}\sum_{k=1}^{n}E_{i,k}^{o⊤}\left({\dot{p}}_{i,k}-{\dot{p}}_{i,k}^{v}\right)=0.\;$$

Consequently, the position channel reduces to

(55) $$\sum_{i=1}^{m}\sum_{k=1}^{n}{\left({\dot{p}}_{i,k}-{\dot{p}}_{i,k}^{v}\right)}^{⊤}E_{i,k}=\sum_{i=1}^{m}\sum_{k=1}^{n}E_{i,k}^{a⊤}\left({\dot{p}}_{i,k}-{\dot{p}}_{i,k}^{v}\right)+\sum_{i=1}^{m}\sum_{k=1}^{n}E_{i,k}^{\epsilon ⊤}\left({\dot{p}}_{i,k}-{\dot{p}}_{i,k}^{v}\right).$$

For the yaw error term, recall from (32) that $e_{i,k}^{\theta }=\theta_{i,k}-\theta_{i,k}^{v}$ and ${\tilde{\omega }}_{i,k}=\omega_{i,k}-{\dot{\theta }}_{i,k}^{v}$. The attitude contribution from (40) reads

(56) $$\sum_{i=1}^{m}\sum_{k=1}^{n}K_{i,k}^{\omega }e_{i,k}^{\theta }\left(\omega_{i,k}-{\dot{\theta }}_{i,k}^{v}\right)=\sum_{i=1}^{m}\sum_{k=1}^{n}K_{i,k}^{\omega }e_{i,k}^{\theta }{\tilde{\omega }}_{i,k}.\;$$

Adding the attitude channel of (48) yields

(57) $$\sum_{i=1}^{m}\sum_{k=1}^{n}K_{i,k}^{\omega }e_{i,k}^{\theta }{\tilde{\omega }}_{i,k}-\sum_{i=1}^{m}\sum_{k=1}^{n}{\tilde{\omega }}_{i,k}\;K_{i,k}^{\omega }e_{i,k}^{\theta }=0.\;$$

Collecting (55) and (57), the guidance-related cross-term from (47) is exactly equivalent to

(58) $$\sum_{i=1}^{m}\sum_{k=1}^{n}{\dot{\tilde{\eta }}}_{i,k}^{⊤}u_{c,i,k}=\sum_{i=1}^{m}\sum_{k=1}^{n}E_{i,k}^{a⊤}\left({\dot{p}}_{i,k}-{\dot{p}}_{i,k}^{v}\right)+\sum_{i=1}^{m}\sum_{k=1}^{n}E_{i,k}^{\epsilon ⊤}\left({\dot{p}}_{i,k}-{\dot{p}}_{i,k}^{v}\right).$$

These two residual terms are identified with $\Xi_{i,k}$ and $Y_{i,k}$ in Stage 4, respectively.

#### 6.4.4. Stage 4: Substitution and Decomposition

Combining the Lyapunov derivative (40) with the kinetic bound (47) and applying the guidance cancellation established in Stage 3, the time derivative of *V* decomposes into five aggregated terms:

(59) $$\dot{V}\leq \sum_{i=1}^{m}\sum_{k=1}^{n}Y_{i,k}+\sum_{i=1}^{m}\sum_{k=1}^{n}\Xi_{i,k}+\sum_{i=1}^{m}\sum_{k=1}^{n}\sum_{\begin{array}{c} l=1\\ l\neq k \end{array}}^{n}\Lambda_{i,k\leftarrow i,l}+\sum_{i=1}^{m}\sum_{k=1}^{n}\sum_{\begin{array}{c} j=1\\ j\neq i \end{array}}^{m}\sum_{l=1}^{n}\Lambda_{i,k\leftarrow j,l}+\sum_{i=1}^{m}\sum_{k=1}^{n}\sum_{o=1}^{\bar{m}}\Gamma_{i,k\leftarrow o},\;$$

where, by virtue of (58),

(60) $$\begin{array}{cc} Y_{i,k} & =-E_{i,k}^{\epsilon ⊤}\left({\dot{p}}_{i,k}-{\dot{p}}_{i,k}^{v}\right), \end{array}$$

(61) $$\begin{array}{cc} \Xi_{i,k} & =E_{i,k}^{a⊤}\left({\dot{p}}_{i,k}-{\dot{p}}_{i,k}^{v}\right), \end{array}$$

(62) $$\begin{array}{cc} \Lambda_{i,k\leftarrow j,l} & =\left(c_{i,k\leftarrow j,l}^{cc}\frac{\partial V_{i,k\leftarrow j,l}^{a}}{\partial p_{i,k}}-E_{i,k\leftarrow j,l}^{a⊤}\right){\dot{p}}_{i,k}, \end{array}$$

(63) $$\begin{array}{cc} \Gamma_{i,k\leftarrow o} & =\left(c_{i,k\leftarrow o}^{cc}\frac{\partial V_{i,k\leftarrow o}^{a}}{\partial p_{i,k}}-E_{i,k\leftarrow o}^{a⊤}\right){\dot{p}}_{i,k}. \end{array}$$

The remainder of the proof proceeds by establishing non-positivity of each aggregated term in (59).

#### 6.4.5. Stage 5: Perturbation Term $Y_{i,k}$

From the deadlock perturbation design (29), the term $Y_{i,k}$ admits two cases:

When $E_{i,k}^{o}=-E_{i,k}^{a}$ with $E_{i,k}^{a}\neq 0$, the perturbation vector satisfies

(64) $$E_{i,k}^{\epsilon }=\frac{\mu_{i,k}^{\epsilon }}{∥E_{i,k}^{a}∥}\;\Theta \;\left(\frac{\pi }{2}\right)E_{i,k}^{a}.\;$$

Since $\Theta \left(\pi /2\right)E_{i,k}^{a}$ is obtained by a π/2 rotation of $E_{i,k}^{a}$, it is orthogonal to $E_{i,k}^{a}$. At the deadlock equilibrium, the velocity error $\left({\dot{p}}_{i,k}-{\dot{p}}_{i,k}^{v}\right)$ is collinear with $E_{i,k}^{a}$ (as both the objective and avoidance forces balance). Hence,

(65) $${\left(\Theta \left(\pi /2\right)E_{i,k}^{a}\right)}^{⊤}\left({\dot{p}}_{i,k}-{\dot{p}}_{i,k}^{v}\right)=0\;\Rightarrow \;Y_{i,k}=0.\;$$

When the deadlock condition does not hold, $E_{i,k}^{\epsilon }=0$ by definition, so $Y_{i,k}=0$ identically.

Consequently,

(66) $$\sum_{i=1}^{m}\sum_{k=1}^{n}Y_{i,k}=0.$$

#### 6.4.6. Stage 6: Virtual Navigation Term $\Xi_{i,k}$

Substituting the virtual navigation velocity (23) into (61) yields

(67) $$\Xi_{i,k}=\left\{\begin{array}{cc} E_{i,k}^{a⊤}\left({\dot{p}}_{i,k}^{d⊥}-{\dot{p}}_{i,k}^{v}\right), & ∥E_{i,k}^{a}∥\neq 0,\\ 0, & ∥E_{i,k}^{a}∥=0. \end{array}\right.\;$$

When avoidance is inactive ($∥E_{i,k}^{a}∥=0$), $\Xi_{i,k}=0$ trivially. When avoidance is active, the perpendicular decomposition (22) ensures $E_{i,k}^{a⊤}{\dot{p}}_{i,k}^{d⊥}=0$. Moreover, during active avoidance, ${\dot{p}}_{i,k}^{v}={\dot{p}}_{i,k}^{d⊥}$ by (23), so the bracketed term vanishes. Therefore,

(68) $$\sum_{i=1}^{m}\sum_{k=1}^{n}\Xi_{i,k}=0.$$

#### 6.4.7. Stage 7: Inter-Agent Avoidance Terms $\Lambda_{i,k\leftarrow j,l}$

This stage treats the intra-formation (j=i, l≠k) and inter-formation (j≠i) terms uniformly. Recall from (18)–(20) that the composite avoidance vector for the pair (i,k)←(j,l) satisfies

(69) $$E_{i,k\leftarrow j,l}^{a⊤}=\left(c_{i,k\leftarrow j,l}^{cc}\;h^{cc}+c_{i,k\leftarrow j,l}^{ac}\;h^{ac}\right)\;\frac{\partial V_{i,k\leftarrow j,l}^{a}}{\partial p_{i,k}},$$

because both $E^{cc}$ and $E^{ac}$ are collinear with ${\left(\partial V/\partial p_{i,k}\right)}^{⊤}$. Substituting (69) into the definition of $\Lambda_{i,k\leftarrow j,l}$ in (62) yields

(70) $$\Lambda_{i,k\leftarrow j,l}=\varrho_{i,k\leftarrow j,l}\;\frac{\partial V_{i,k\leftarrow j,l}^{a}}{\partial p_{i,k}}\;{\dot{p}}_{i,k},\;$$

where the scalar coefficient $\varrho_{i,k\leftarrow j,l}$ is defined as

(71) $$\varrho_{i,k\leftarrow j,l}≜c_{i,k\leftarrow j,l}^{cc}-c_{i,k\leftarrow j,l}^{cc}\;h^{cc}\;\left(1-\frac{{\dot{V}}_{i,k\leftarrow j,l}^{a}}{\alpha_{i,k\leftarrow j,l}}\right)-c_{i,k\leftarrow j,l}^{ac}\;h^{ac}\left({\dot{V}}_{i,k\leftarrow j,l}^{a}\right).\;$$

To symmetrize the double sum, consider the reverse ordered pair (j,l)←(i,k). By the antisymmetry of the spatial gradient,

(72) $$\frac{\partial V_{j,l\leftarrow i,k}^{a}}{\partial p_{j,l}}=-\frac{\partial V_{i,k\leftarrow j,l}^{a}}{\partial p_{i,k}},\;$$

and by the weight symmetry $c_{j,l\leftarrow i,k}^{cc}=c_{i,k\leftarrow j,l}^{cc}$, $c_{j,l\leftarrow i,k}^{ac}=c_{i,k\leftarrow j,l}^{ac}$ together with the fact that $h^{cc}$ and $h^{ac}$ depend only on the symmetric quantities φ and $\dot{V}$, we obtain

(73) $$\Lambda_{j,l\leftarrow i,k}=-\varrho_{i,k\leftarrow j,l}\;\frac{\partial V_{i,k\leftarrow j,l}^{a}}{\partial p_{i,k}}\;{\dot{p}}_{j,l}.\;$$

Adding (70) and (73) gives the pairwise identity

(74) $$\Lambda_{i,k\leftarrow j,l}+\Lambda_{j,l\leftarrow i,k}=\varrho_{i,k\leftarrow j,l}\;\frac{\partial V_{i,k\leftarrow j,l}^{a}}{\partial p_{i,k}}\;\left({\dot{p}}_{i,k}-{\dot{p}}_{j,l}\right)=\varrho_{i,k\leftarrow j,l}\;{\dot{V}}_{i,k\leftarrow j,l}^{a},$$

where the last equality follows from the chain rule and the gradient antisymmetry (72). Summing over all ordered pairs and exploiting the pairwise cancellation (74), we arrive at the symmetrized expression

(75) $$\sum_{i,k}\sum_{j,l}\Lambda_{i,k\leftarrow j,l}=\frac{1}{2}\sum_{i,k}\sum_{j,l}\varrho_{i,k\leftarrow j,l}\;{\dot{V}}_{i,k\leftarrow j,l}^{a}.\;$$

The sign of each summand $\varrho_{i,k\leftarrow j,l}{\dot{V}}_{i,k\leftarrow j,l}^{a}$ is determined by the relative motion regime, which partitions into four disjoint regions:

Case 1: Outside detection zone: $\varphi_{i,k\leftarrow j,l}>\psi_{i,k\leftarrow j,l}$. Then, $V_{i,k\leftarrow j,l}^{a}\equiv 0$ and ${\dot{V}}_{i,k\leftarrow j,l}^{a}\equiv 0$, so $\varrho_{i,k\leftarrow j,l}{\dot{V}}_{i,k\leftarrow j,l}^{a}=0$.
Case 2: Safety region: ${\dot{V}}_{i,k\leftarrow j,l}^{a}\leq \alpha_{i,k\leftarrow j,l}<0$. Here, $h^{cc}=h^{ac}=0$, yielding $\varrho_{i,k\leftarrow j,l}=c_{i,k\leftarrow j,l}^{cc}>0$. Since ${\dot{V}}_{i,k\leftarrow j,l}^{a}<0$,
  (76) $$\varrho_{i,k\leftarrow j,l}{\dot{V}}_{i,k\leftarrow j,l}^{a}<0.\;$$
Case 3: Transition region: $\alpha_{i,k\leftarrow j,l}<{\dot{V}}_{i,k\leftarrow j,l}^{a}\leq 0$. Here, $h^{ac}=0$ and $0<h^{cc}\leq 1$, so $0\leq \varrho_{i,k\leftarrow j,l}<c_{i,k\leftarrow j,l}^{cc}$. Together with ${\dot{V}}_{i,k\leftarrow j,l}^{a}\leq 0$,
  (77) $$\varrho_{i,k\leftarrow j,l}{\dot{V}}_{i,k\leftarrow j,l}^{a}\leq 0.\;$$
Case 4: Conflict region: ${\dot{V}}_{i,k\leftarrow j,l}^{a}>0$. Here, $h^{cc}=1$ and $h^{ac}={\dot{V}}_{i,k\leftarrow j,l}^{a}$, giving
  (78) $$\varrho_{i,k\leftarrow j,l}=-c_{i,k\leftarrow j,l}^{ac}{\dot{V}}_{i,k\leftarrow j,l}^{a}.\;$$
  Substituting (78) into (75), we obtain
  (79) $$\sum_{i,k}\sum_{j,l}\Lambda_{i,k\leftarrow j,l}=-\frac{1}{2}\sum_{i,k}\sum_{j,l}c_{i,k\leftarrow j,l}^{ac}{\left({\dot{V}}_{i,k\leftarrow j,l}^{a}\right)}^{2}\leq 0.\;$$

In all regimes, $\varrho_{i,k\leftarrow j,l}{\dot{V}}_{i,k\leftarrow j,l}^{a}\leq 0$. Therefore,

(80) $$\sum_{i=1}^{m}\sum_{k=1}^{n}\sum_{\begin{array}{c} l=1\\ l\neq k \end{array}}^{n}\Lambda_{i,k\leftarrow i,l}+\sum_{i=1}^{m}\sum_{k=1}^{n}\sum_{\begin{array}{c} j=1\\ j\neq i \end{array}}^{m}\sum_{l=1}^{n}\Lambda_{i,k\leftarrow j,l}\leq 0.$$

#### 6.4.8. Stage 8: Obstacle Avoidance Terms $\Gamma_{i,k\leftarrow o}$

By the same four-case analysis as in Stage 7 (outside detection, safety, transition, and conflict), one verifies that $\varrho_{i,k\leftarrow o}{\dot{V}}_{i,k\leftarrow o}^{a}\leq 0$ in all cases. Consequently,

(81) $$\sum_{i=1}^{m}\sum_{k=1}^{n}\sum_{o=1}^{\bar{m}}\Gamma_{i,k\leftarrow o}\leq 0.\;$$

#### 6.4.9. Stage 9: Non-Positivity of $\dot{V}$

Substituting (66), (68), (80), and (81) into the decomposed derivative (59), and recalling the dissipation term from (47), we obtain the consolidated inequality

(82) $$\dot{V}\leq 0,\;$$

which establishes that V(t) is non-increasing and uniformly bounded above by V(0) for all t≥0.

#### 6.4.10. Stage 10: Safety Guarantee (Property P1)

We proceed by contradiction. Suppose a collision occurs at some finite time $t_{c}>0$. Then, there exists at least one pair (i,k),(j,l) or an agent–obstacle pair (i,k),o such that

(83) $$\varphi_{i,k\leftarrow j,l}\left(t\right)\to \gamma_{i,k\leftarrow j,l}^{+}\;\mathrm{or}\;\varphi_{i,k\leftarrow o}\left(t\right)\to \gamma_{i,k\leftarrow o}^{+}.\;$$

By the barrier construction (16), this implies $V_{i,k\leftarrow j,l}^{a}\left(t\right)\to +\infty$ (resp. $V_{i,k\leftarrow o}^{a}\left(t\right)\to +\infty$), which drives V(t)→+∞. However, the consolidated inequality $\dot{V}\leq 0$ established in Stage 9 ensures V(t)≤V(0)<+∞ for all t≥0, yielding a contradiction. Therefore, no collision can occur, and Property (P1) holds.

#### 6.4.11. Stage 11: Position Convergence (Property P2)

From (82), $\dot{V}\leq 0$ and V(t) is bounded. By LaSalle’s invariance principle for non-smooth systems [39], the trajectories converge to the largest invariant set contained in $\left\{\dot{V}=0\right\}$. The condition $\dot{V}=0$ requires ${\dot{\tilde{\eta }}}_{i,k}=0$ for all i,k, which implies $\omega_{i,k}={\dot{\theta }}_{i,k}^{v}$ and, from the kinematic relation ${\dot{p}}_{i,k}=R_{i,k}v_{i,k}$ together with the above, we obtain

(84) $${\dot{p}}_{i,k}={\dot{p}}_{i,k}^{v}-E_{i,k}.\;$$

At the invariant set, ${\dot{\tilde{\eta }}}_{i,k}=0$ also implies ${\dot{p}}_{i,k}={\dot{p}}_{i,k}^{v}$ (since ${\dot{\tilde{\eta }}}_{i,k}=0$ means the actual velocity equals the desired velocity ${\dot{\eta }}_{i,k}^{d}={\left[{\left({\dot{p}}_{i,k}^{v}\right)}^{⊤},{\dot{\theta }}_{i,k}^{v}\right]}^{⊤}$). Equating the two expressions for ${\dot{p}}_{i,k}$ gives $E_{i,k}=0$. In the invariant set, the avoidance and perturbation vectors are inactive ($E_{i,k}^{a}=0$, $E_{i,k}^{\epsilon }=0$), whence $E_{i,k}^{o}=0$. By the definition of $E_{i,k}^{o}$ in (28), $E_{i,k}^{o}=0$ if and only if $e_{i,k}=0$. Hence,

(85) $$\lim_{t\to \infty }∥p_{i,k}\left(t\right)-p_{i,k}^{v}\left(t\right)∥=0,\;\forall i,k,$$

establishing Property (P2).

## 7. Comparative Simulations

To validate the effectiveness of the proposed decentralized closed-form control strategy, we conduct comparative simulations against a traditional artificial potential field (APF) method that relies solely on position information (pos-only). The simulation scenario involves two formations (Formation 1 and Formation 2), each consisting of three UAVs, navigating in a cluttered environment with a central circular obstacle.

### 7.1. Simulation Setup

The simulation is conducted with six UAVs governed by a 3-DoF kinematic model with inner-loop attitude stabilization (Section 2.2), organized into two triangular formations specified by relative position vectors $\delta_{i,k}$ and orientation offsets $\psi_{i,k}$ with respect to virtual centroids whose trajectories are generated by the proposed virtual navigation strategy (Section 4). A single circular obstacle of radius R=40 m is placed at the origin, with barrier potential parameters μ=2, $\psi_{i,k\leftarrow o}=50\;m$, $\gamma_{i,k\leftarrow o}=42\;m$, and event-triggering threshold $\alpha_{i,k\leftarrow o}=-0.5$. The proposed controller employs velocity-informed event-triggering with blending weights $c^{cc}=1$, $c^{ac}=1.2$, convergence gain $K_{i,k}=\mathrm{diag}\left(2,2,1.5\right)$, and disturbance bound ${\bar{d}}_{i,k}=0.1$. The six UAVs are initialized in two formations approaching from orthogonal directions: Formation 1 (UAVs 1–3) starts from the south at (0,−150), (20,−170), and (−20,−170) with heading θ=π/2, while Formation 2 (UAVs 4–6) starts from the west at (−150,−10), (−170,10), and (−170,−30) with heading θ=0, thereby creating a challenging multi-formation collision avoidance scenario. Two methods are compared: the proposed method, which incorporates both position and velocity information via event-triggered generalized *p*-norm distance assessment (p=2, $\Phi =I_{2}$ for circular obstacles); and the traditional APF, which uses standard Euclidean-distance-based repulsive forces activated purely by geometric proximity without velocity prediction. All simulations are executed in MATLAB/Simulink 2022a with a fixed-step solver (step size 0.01 s) over a time horizon of 300 s.

**Remark** **3** (Controlled Comparison)**.** *It is noteworthy that our comparison is designed as a controlled study isolating the impact of the event-triggered mechanism. Specifically, the proposed method is constructed directly upon the traditional artificial potential field (APF) framework by augmenting it with velocity-informed event-triggered activation, while preserving the closed-form structure. Consequently, the baseline APF serves as the natural reference: both methods share the same underlying potential-field architecture and closed-form computational nature, differing only in whether repulsive forces are continuously activated by geometric proximity or selectively triggered by increasing collision risk. Methods that depart from the APF framework or rely on real-time iterative optimization, such as model predictive control (MPC), distributed MPC, and CBF-based quadratic programming, were not included in our direct comparison, as they lack closed-form representations and would introduce confounding variables (e.g., optimization horizons, iteration tolerances) that preclude a fair attribution of performance differences to the event-triggering mechanism alone.*

### 7.2. Trajectory Analysis

**Figure 4:**
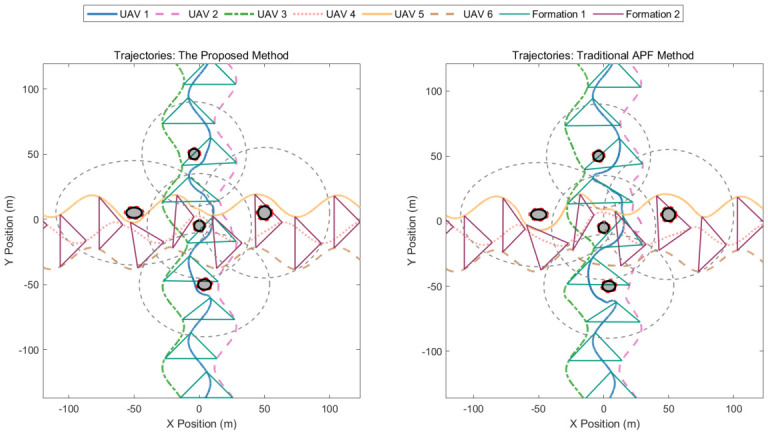
Spatial trajectories of two formations (six UAVs total) navigating around a central obstacle. Left: The proposed method with event-triggered avoidance. Right: Traditional APF method. Formation 1 (UAVs 1–3, blue/magenta/green) approaches from the south; Formation 2 (UAVs 4–6, dotted pink/orange/dashed brown) approaches from the west. The obstacle is located at the origin with radius R=40 m. The proposed method exhibits smooth, coordinated trajectories with preserved formation geometry, while the traditional APF method shows oscillatory paths and trajectory crossovers near the obstacle boundary.

Figure 4 presents the spatial trajectories of all six UAVs for both methods. The proposed method (left panel) demonstrates smooth, coordinated trajectories with the two formations successfully navigating around the central obstacle while maintaining their geometric configurations. UAVs 1–3 approach from the south, execute a coordinated leftward evasion, and smoothly transit to their target region. UAVs 4–6 approach from the west, perform a symmetric rightward evasion, and rejoin their formation geometry. The virtual-centroid-based architecture ensures that the triangular formation shapes are preserved throughout the maneuver, with UAV trajectories exhibiting continuous curvature and no crossovers.

In contrast, the traditional APF method (right panel) exhibits severe oscillatory trajectories near the obstacle. In particular, UAV 4 (dotted pink) undergoes pronounced chattering and trajectory crossover around the obstacle boundary, while UAVs 1 and 2 also display noticeable winding paths. The lack of velocity-informed triggering causes premature activation of repulsive forces, resulting in trajectory instability and significantly degraded formation coherence. Individual UAVs execute unnecessary evasive maneuvers even when the collision risk is non-increasing, leading to large deviations from the nominal formation geometry.

### 7.3. Safety Margin Evaluation

**Figure 5:**
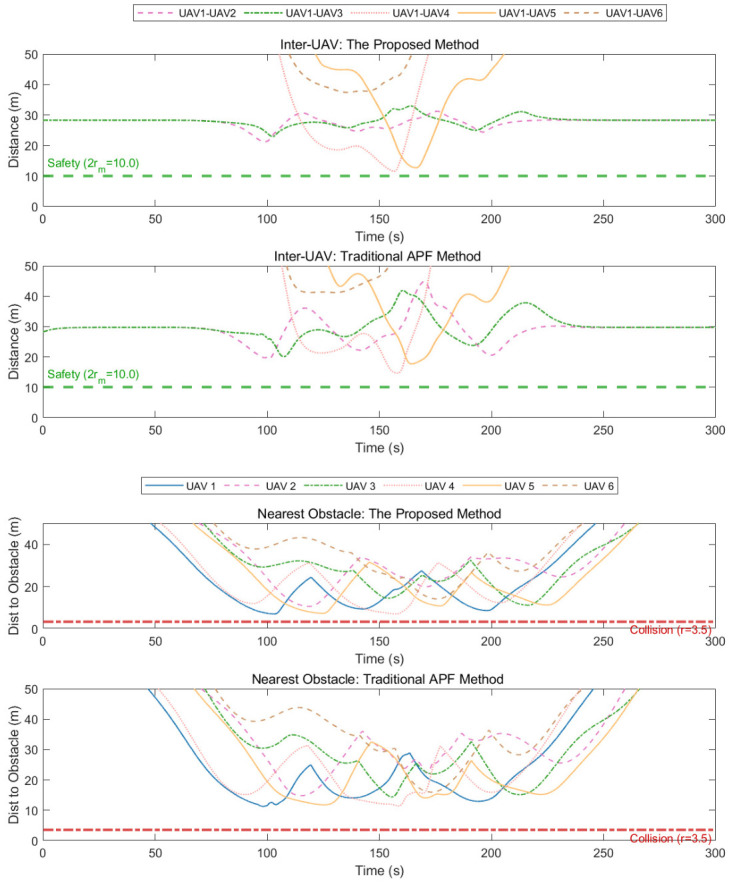
Safety margin comparison. Top two panels: Inter-UAV distances for the proposed method (upper) and traditional APF (lower). The green dashed line denotes the safety threshold ($2r_{m}=10.0$ m). Bottom two panels: Nearest obstacle distances. The red dashed line denotes the collision radius (r=3.5 m). The proposed method maintains tighter yet safe clearance, while the traditional APF exhibits excessive conservatism with large-distance fluctuations.

Figure 5 illustrates the inter-UAV distances and agent-to-obstacle distances over time. For inter-UAV safety (top two panels), the proposed method maintains all inter-UAV distances strictly above the collision threshold $2r_{m}=10.0$ m throughout the simulation, with a minimum recorded distance of 12.0 m, demonstrating adequate safety preservation with moderate conservatism. In contrast, the traditional APF achieves a minimum inter-UAV distance of 15.0 m, which appears superficially safer but reflects excessive conservatism; specifically, the inter-UAV distances exhibit large-amplitude fluctuations ranging from 15 m to over 50 m, indicating that the position-only activation criterion causes premature repulsive responses, forcing agents to maintain unnecessarily large and highly variable separations that degrade formation compactness and mission efficiency. Regarding obstacle avoidance (bottom two panels), the proposed method keeps all UAVs above the obstacle collision radius r=3.5 m with a minimum distance of 7.2 m, confirming successful collision avoidance with balanced risk assessment; the velocity-informed triggering allows UAVs to approach closer to the obstacle when the risk is decreasing, thereby optimizing path efficiency. Conversely, the traditional APF yields a minimum obstacle distance of 12.0 m, indicating excessive conservatism as the position-only mechanism activates repulsive forces prematurely, preventing optimal trajectory planning and resulting in longer path lengths and higher energy consumption.

### 7.4. Kinematic Performance Profiles

**Figure 6:**
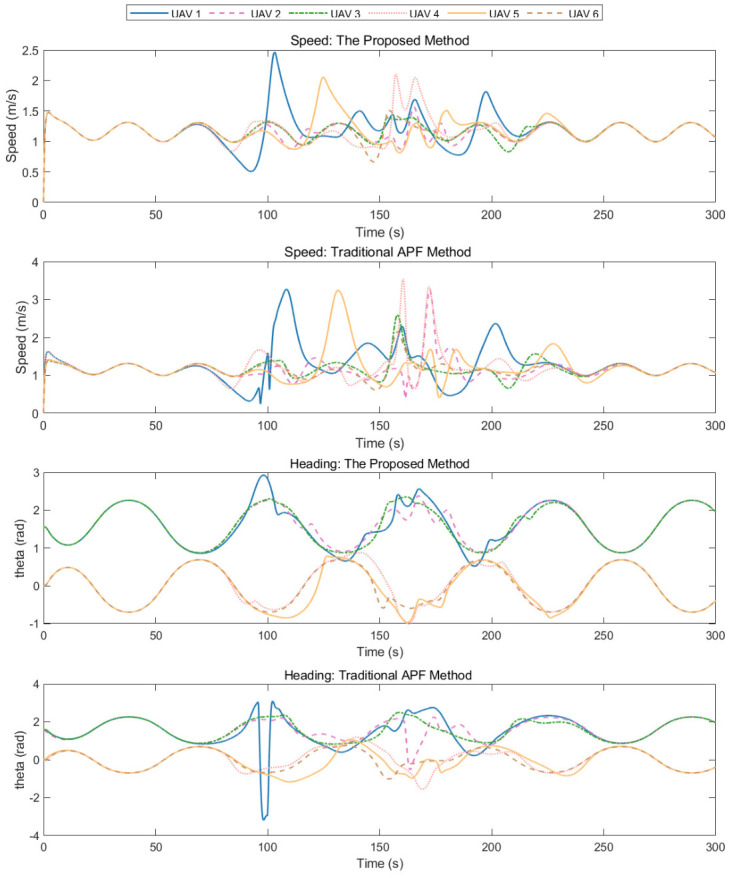
Kinematic profiles comparison. Top two panels: Speed evolution. The proposed method (upper) shows smooth transitions with a peak of 2.5 m/s, while the traditional APF (lower) exhibits severe oscillations reaching 3.5 m/s. Bottom two panels: Heading angle evolution. The proposed method maintains continuous heading profiles, whereas the traditional APF suffers from drastic jumps (e.g., UAV 1 crossing the ±π boundary near t=100 s).

Figure 6 presents the speed and heading angle profiles for all UAVs. Regarding speed (top two panels), the proposed method achieves a maximum flight speed of 2.5 m/s (UAV 1 at t≈100 s), with smooth transitions during the avoidance phase (t∈[100,200] s); the velocity-informed event triggering prevents abrupt decelerations or accelerations, thereby preserving energy efficiency and actuator longevity. In contrast, the traditional APF reaches a maximum flight speed of 3.5 m/s, representing a 40% increase over the proposed method, where these speed spikes indicate control chattering and excessive energy expenditure caused by the continuous activation of repulsive potentials without velocity-based modulation, and the large speed variations also impose significant stress on the inner-loop controllers and physical actuators. For heading angles (bottom two panels), the proposed method produces smoothly evolving profiles with continuous derivatives, and the maximum heading rate during avoidance is well-controlled, ensuring dynamic feasibility and preventing saturation of the yaw actuators. Conversely, the traditional APF exhibits severe discontinuities and rapid oscillations; most notably, UAV 1 (blue solid) undergoes a drastic jump from approximately 2.5 rad to nearly −3 rad around t=100 s, crossing the phase-wrap boundary, while UAV 4 (dotted pink) also exhibits large heading fluctuations around t=150 s. These instabilities reflect the competing attractive and repulsive forces in the potential field and correlate directly with the speed spikes observed in the top panel, and such heading discontinuities are highly undesirable in practice as they can induce actuator saturation and attitude-control failures.

### 7.5. Avoidance Control Effort

**Figure 7:**
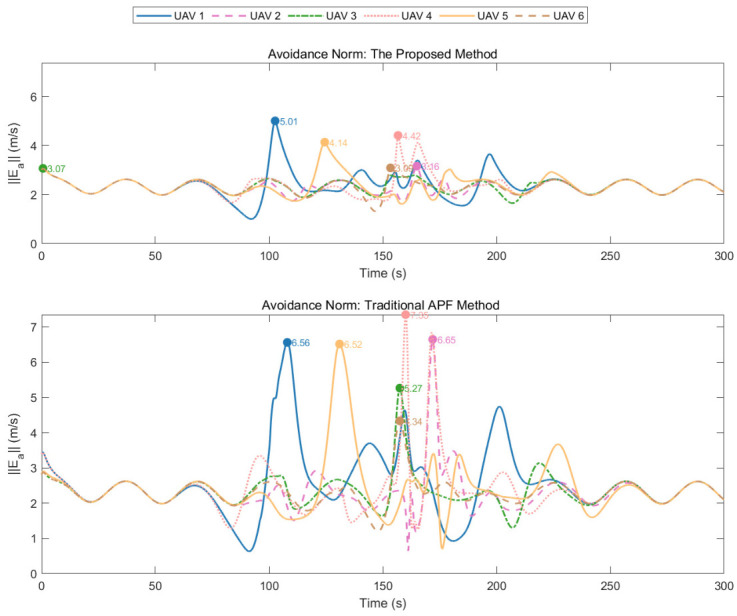
Avoidance norm $∥E_{i,k}^{a}∥$ comparison. Top: The proposed method with velocity-informed event-triggering. Peak values: UAV 1 (5.01), UAV 2 (3.36), UAV 3 (3.07), UAV 4 (4.14), UAV 5 (4.42), UAV 6 (3.26). Bottom: Traditional APF with continuous activation. Peak values: UAV 1 (6.56), UAV 2 (5.34), UAV 3 (5.27), UAV 4 (6.52), UAV 5 (7.35), UAV 6 (6.65). The event-triggered mechanism reduces both peak magnitudes and activation duration.

Figure 7 compares the norm of the avoidance vector $∥E_{i,k}^{a}∥$ for both methods.

Proposed Method: The avoidance control effort peaks at moderate values (maximum 5.01 for UAV 1 at t≈100 s), with other agents reaching 4.14 (UAV 4), 4.42 (UAV 5), 3.36 (UAV 2), 3.26 (UAV 6), and 3.07 (UAV 3). The effort decays rapidly once the collision risk decreases. The event-triggered mechanism ensures that avoidance forces are active only when ${\dot{V}}_{i,k\leftarrow j,l}^{a}>0$, significantly reducing cumulative control effort.

Traditional APF: The avoidance norm exhibits higher peaks and prolonged activation periods. The global maximum reaches 7.35 (UAV 5), with multiple agents simultaneously experiencing high effort exceeding 5.0, including UAV 1 (6.56), UAV 4 (6.52), and UAV 3 (5.27). The position-only activation criterion causes unnecessary repulsive forces even when agents are moving away from each other, resulting in synchronized chattering across the team and significantly higher cumulative avoidance effort compared to the proposed method.

### 7.6. Quantitative Performance Metrics

Table 2 summarizes the quantitative comparison across key performance indicators.

Key Observations: While the APF method records larger minimum distances (inter-UAV: 15.0 m vs. 12.0 m; obstacle: 12.0 m vs. 7.2 m), this reflects excessive conservatism rather than superior safety, as the proposed method maintains safe margins while optimizing path efficiency, whereas the APF wastes maneuvering space and energy through premature avoidance activation. In terms of dynamic performance, the proposed method achieves a substantially lower peak speed (2.5 m/s vs. 3.5 m/s), directly translating to lower actuator stress, reduced energy consumption, and improved flight stability, and more importantly, it eliminates the severe heading discontinuities observed in the APF method, which is critical for physical actuator safety. Regarding efficiency, the proposed method exhibits shorter cumulative avoidance time (30 s vs. 56 s) and lower energy cost, confirming that it avoids unnecessary evasive maneuvers since the velocity-informed triggering ensures that avoidance forces are injected only when collision risk is genuinely increasing. Finally, the closed-form control law maintains a per-step computational cost of 0.8 ms, achieved by evaluating elementary algebraic functions without invoking online numerical optimization, which confirms the lightweight computational nature of the proposed architecture.

### 7.7. Discussion

The simulation results validate the theoretical guarantees established in Theorem 1. The proposed virtual-centroid-based architecture successfully eliminates leader dependency while preserving formation geometry through distributed coordination. The integration of velocity information into the event-triggered mechanism addresses the fundamental limitation of traditional APF methods: the inability to distinguish between approaching and receding collision risks.

The deadlock perturbation vector (Section 5.1.2) plays a critical role in the obstacle-rich region, ensuring that UAVs do not become trapped in local equilibria where attractive and repulsive forces balance. The virtual navigation trajectory (Section 4) enables seamless coordination between tracking and avoidance without the trajectory freezing artifacts observed in conventional priority-based scheduling.

The generalized *p*-norm distance function (Section 3) contributes to the reduced conservatism by providing geometrically accurate collision risk assessment, while the current simulation uses circular obstacles (p=2, $\Phi =I_{2}$), the framework is readily extensible to elliptical, rectangular, and non-convex geometries without structural modifications.

A particularly noteworthy observation from the new results is the severe heading instability exhibited by the traditional APF method. The abrupt heading jumps (e.g., UAV 1 crossing the ±π boundary near t=100 s) demonstrate that position-only potential fields can generate physically infeasible yaw commands, whereas the proposed method guarantees smooth heading transitions essential for real-world UAV operations.

## 8. Conclusions

This paper presents a distributed closed-form control framework for multi-UAV formation flight that simultaneously addresses formation tracking, collision avoidance, and virtual-centroid navigation. By introducing a virtual-centroid-based architecture, the proposed method reduces the single-point-of-failure vulnerability inherent in leader–follower schemes while preserving precise geometric configuration through coordinated local feedback. The virtual navigation trajectory dynamically integrates avoidance information with reference tracking, enabling autonomous coordination of tracking and collision avoidance without priority-based scheduling or trajectory freezing artifacts.

A generalized *p*-norm distance function is developed to accurately assess collision risks for elliptical, rectangular, rhombic, and non-convex star-shaped obstacles, significantly reducing the conservatism of Euclidean approximations. Complementing this, an event-triggered collision avoidance mechanism with velocity-dependent activation and CBF-inspired constraints produces amplitude-modulated, continuity-preserving control vectors that suppress chattering and avoid unnecessary evasive maneuvers.

Lyapunov-based analysis guarantees collision avoidance (forward invariance of safe sets) and asymptotic position convergence to the desired formation. Comparative simulations demonstrate that the proposed method achieves smooth trajectories with efficient collision avoidance through lightweight closed-form computation, while reducing cumulative avoidance time and energy consumption relative to conventional APF methods. The closed-form control law maintains constant per-agent computational complexity per step, ensuring graceful scaling to larger team sizes under mild communication connectivity assumptions.

Future work will extend the framework to full six-degree-of-freedom (6-DoF) aerial vehicles operating in three-dimensional cluttered environments, investigate learning-based virtual navigation strategies, and explore resilient coordination under malicious cyber-physical attacks and communication denial scenarios.

## Figures and Tables

**Table 1:** Summary of Unified Notation and Signal-Flow Hierarchy.

Symbol	Description
(a) Index Convention	
$i,j\in N_{1}^{m}$	Formation indices
$k,l\in N_{1}^{n}$	Agent indices within a formation
$o\in N_{1}^{\bar{m}}$	Obstacle index
(b) State Variables	
$\eta_{i,k}={\left[x_{i,k},y_{i,k},\theta_{i,k}\right]}^{⊤}\in R^{3}$	Generalized coordinates (inertial frame)
$\nu_{i,k}={\left[v_{i,k}^{⊤},\omega_{i,k}\right]}^{⊤}\in R^{3}$	Generalized velocity (body-fixed frame)
$p_{i,k}={\left[x_{i,k},y_{i,k}\right]}^{⊤}\in R^{2}$	Planar position (extracted from $\eta_{i,k}$)
$\theta_{i,k}\in \left(-\pi ,\pi \right]$	Yaw angle (shared with dynamics)
${\dot{\tilde{\eta }}}_{i,k}={\dot{\eta }}_{i,k}-{\dot{\eta }}_{i,k}^{d}\in R^{3}$	Generalized velocity error (earth-fixed)
${\tilde{\nu }}_{i,k}=\nu_{i,k}-J_{i,k}^{-1}{\dot{\eta }}_{i,k}^{d}\in R^{3}$	Body-frame velocity error
$e_{i,k}^{\theta }=\theta_{i,k}-\theta_{i,k}^{v}\in R$	Yaw tracking error
(c) Signal-Flow Hierarchy (E→v→τ)	
$E_{i,k}=-E_{i,k}^{o}+E_{i,k}^{a}+E_{i,k}^{\epsilon }$	Composite guidance vector (inertial frame)
$E_{i,k}^{o}$	Formation objective vector (attractive)
$E_{i,k}^{a}$	Collision avoidance vector (repulsive)
$E_{i,k}^{\epsilon }$	Deadlock perturbation vector
$u_{i,k}={\left[{\left(u_{i,k}^{v}\right)}^{⊤},u_{i,k}^{\omega }\right]}^{⊤}\in R^{3}$	Generalized control input (body frame)
$\tau_{i,k}^{\eta }=J_{i,k}^{-⊤}\tau_{i,k}$	Earth-fixed equivalent actuation
$u_{c,i,k}={\left[E_{i,k}^{⊤},-K_{i,k}^{\omega }e_{i,k}^{\theta }\right]}^{⊤}$	Guidance-related input (earth-fixed)
$\Lambda_{i,k},\;\Lambda_{i,k}^{\eta }$	Disturbance rejection terms (body/earth-fixed)
${\ddot{\eta }}_{i,k}^{d}$	Desired generalized acceleration
(d) Virtual-Structure Variables	
$p_{i}^{c}\in R^{2}$	Virtual centroid position of formation *i*
$\theta_{i}^{c}\in \left(-\pi ,\pi \right]$	Virtual centroid orientation
$p_{i,k}^{d},\theta_{i,k}^{d}$	Desired position/orientation w.r.t. centroid
$p_{i,k}^{v},\theta_{i,k}^{v}$	Virtual navigation position/angle
$e_{i,k}=p_{i,k}-p_{i,k}^{v}$	Position tracking error (classic form)
(e) Safety Functions	
$V_{i,k\leftarrow j,l}^{a},\;V_{i,k\leftarrow o}^{a}$	Avoidance functions (smooth, compact support)
$h_{i,k\leftarrow j,l},\;h_{i,k\leftarrow o}$	Safety margins (forward invariance)
$\varphi_{i,k\leftarrow j,l},\;\varphi_{i,k\leftarrow o}$	Generalized *p*-norm distance functions
$\psi_{i,k\leftarrow j,l},\;\psi_{i,k\leftarrow o}$	Detection/activation boundaries
$\gamma_{i,k\leftarrow j,l},\;\gamma_{i,k\leftarrow o}$	Safety margins (collision boundaries)
$\alpha_{i,k\leftarrow j,l},\;\alpha_{i,k\leftarrow o}$	Event-triggering thresholds (α<0)
$h^{cc}\left(\;\cdot \;\right),\;h^{ac}\left(\;\cdot \;\right)$	Continuity-control and amplitude-control step functions
$c^{cc},\;c^{ac}$	Blending weights for continuity and amplitude control
(f) System and Control Parameters	
$M_{i,k}\in R^{3\times 3}$	Inertia matrix (body-fixed)
$m_{i,k},I_{i,k}$	Equivalent mass and yaw inertia
$d_{i,k}={\left[{\left(d_{i,k}^{v}\right)}^{⊤},d_{i,k}^{\omega }\right]}^{⊤}$	External disturbance, $∥d_{i,k}∥\leq {\bar{d}}_{i,k}$
$K_{i,k}^{\omega }>0$	Yaw control gain
${\bar{d}}_{i,k}>0$	Known disturbance upper bound

**Table 2:** Quantitative Performance Comparison.

Metric	Proposed	Traditional APF
Minimum Inter-UAV Distance [m]	12.0	15.0
Minimum Obstacle Distance [m]	7.2	12.0
Maximum Flight Speed [m/s]	2.5	3.5
Speed Variation (Std. Dev.) [m/s]	0.22	0.48
Peak Avoidance Effort	5.01	7.35
Cumulative Avoidance Time [s]	30	56
Energy Cost Proxy ($\int ∥\dot{p}{∥}_{Q}^{2}dt$) [$10^{3}$]	1.35	2.15
Computational Time per Step [ms]	0.8	0.6

## Data Availability

Data are contained within the article.
